# Reducing RBM20 activity improves diastolic dysfunction and cardiac atrophy

**DOI:** 10.1007/s00109-016-1483-3

**Published:** 2016-11-26

**Authors:** Florian Hinze, Christoph Dieterich, Michael H. Radke, Henk Granzier, Michael Gotthardt

**Affiliations:** 1 Neuromuscular and Cardiovascular Cell Biology, Max Delbrück Center for Molecular Medicine, Robert-Rössle-Str. 10, 13125 Berlin, Germany; 2 DZHK (German Center for Cardiovascular Research), partner site Berlin, Berlin, Germany; 3 Klaus Tschira Institute for Integrative Computational Cardiology and Department of Cardiology, Angiology, and Pneumology, Heidelberg University, Analysezentrum III, INF 669, 69120 Heidelberg, Germany; 4 DZHK (German Center for Cardiovascular Research), partner site Heidelberg, Heidelberg, Germany; 5 Department of Cellular and Molecular Medicine, University of Arizona, Arizona Health Sciences Center, 1501 N. Campbell, PO Box 245051, Tucson, AZ 85724 USA

**Keywords:** Heart failure, Therapy, Mouse models, RNA processing, Hypertrophy signaling

## Abstract

**Abstract:**

Impaired diastolic filling is a main contributor to heart failure with preserved ejection fraction (HFpEF), a syndrome with increasing prevalence and no treatment. Both collagen and the giant sarcomeric protein titin determine diastolic function. Since titin’s elastic properties can be adjusted physiologically, we evaluated titin-based stiffness as a therapeutic target. We adjusted RBM20-dependent cardiac isoform expression in the titin N2B knockout mouse with increased ventricular stiffness. A ~50 % reduction of RBM20 activity does not only maintain cardiac filling in diastole but also ameliorates cardiac atrophy and thus improves cardiac function in the N2B-deficient heart. Reduced RBM20 activity partially normalized gene expression related to muscle development and fatty acid metabolism. The adaptation of cardiac growth was related to hypertrophy signaling via four-and-a-half lim-domain proteins (FHLs) that translate mechanical input into hypertrophy signals. We provide a novel link between cardiac isoform expression and trophic signaling via FHLs and suggest cardiac splicing as a therapeutic target in diastolic dysfunction.

**Key message:**

- Increasing the length of titin isoforms improves ventricular filling in heart disease.
- FHL proteins are regulated via RBM20 and adapt cardiac growth.
- RBM20 is a therapeutic target in diastolic dysfunction.

**Electronic supplementary material:**

The online version of this article (doi:10.1007/s00109-016-1483-3) contains supplementary material, which is available to authorized users.

## Introduction

Cardiovascular disease is the main cause of death worldwide with increasing prevalence of heart failure [1]. Multiple environmental and genetic factors contribute to heart failure including age, sex, diabetes, kidney disease, inflammation, and mutations in sarcomeric proteins such as titin or cardiac splice factors such as the RNA binding motif 20 (RBM20) that regulates titin-based stiffness [2]. The giant sarcomeric protein titin contributes to the diastolic properties of the heart. Titin undergoes extensive posttranslational modifications and alternative splicing adapts its elastic properties to the demands of the organism [3, 4]. The elastic PEVK and N2B regions support diastolic function, while differentially affecting cardiac growth [5, 6]. The PEVK region serves as an entropic spring, while the N2B region improves efficiency of the cardiac cycle via altered calcium sensitivity [7, 8]. Changes in titin isoform expression relate primarily to the elastic PEVK, N2B, and inter-adjacent immunoglobulin (IG) regions and are mediated by RBM20, the first splice factor related to human heart disease [9]. Patients with mutations in Rbm20 express more compliant titin isoforms associated with dilated cardiomyopathy, fibrosis, and sudden cardiac death [9, 10]. Both, a naturally occurring RBM20-deficient rat strain and mice carrying a deletion of the RBM20 RNA recognition motif (RRM), express similar giant titin isoforms and recapitulate human RBM20 deficiency [2, 11].

In mice, increased diastolic compliance associated with longer titin isoforms contributes to improved cardiac function [11]. In patients, the shift in titin isoform-expression from the stiff N2B to the more compliant N2BA isoform is also associated with improved function [4, 12]. This change in titin-based elasticity compensates for the increased ventricular stiffness by fibrosis. To evaluate if cardiac splicing could serve as a therapeutic target to decrease titin-based stiffness and could provide a lasting beneficial effect on diastolic dysfunction, we crossed the splice-deficient Rbm20^∆RRM^ mouse and the titin N2B knockout mouse (Titin N2B^−/−^). Excision of the elastic N2B element of titin affects the mechanical properties of the sarcomere, hypertrophy signaling, and ultimately leads to a restrictive filling pattern [5]. In double-deficient mice (Titin^∆N2B/∆N2B^ Rbm20^∆RRM/WT^), reduced splicing with expression of more compliant titin isoforms had several positive effects. Not only was diastolic compliance improved but also cardiac dimensions, RNA levels of genes related to the cAMP response, and oxidative phosphorylation were restored. These findings suggest that RBM20 could be a therapeutic target in diastolic dysfunction.

## Materials and methods

### Animal procedures

Mice were sacrificed by cervical dislocation at 100 to 120 days of age. The hearts were rapidly excised, washed in PBS, and dissected into atria, septum, right and left ventricle, and tissues were snap frozen in liquid nitrogen and stored at −80 °C. Mice were age and sex matched for each analysis (100–120 days old males). Generation of animal models and phenotyping by histology, echocardiograpy and conductance catheter is described in the supplement. All experiments involving animals were carried out following the Guide for the Care and Use of Laboratory Animals of the German animal welfare act and protocols were approved by the Committee on the Ethics of Animal Experiments of Berlin State authorities (LaGeSo).

### Analysis and quantification of RNA and protein expression

Proteins and RNA were extracted from left ventricular tissue powder. Proteins were separated and blotted as described previously [2]. Antibodies were used according to manufacturer’s instructions and are listed in Supplemental Table 1. Analysis of titin isoform expression was performed as described previously [13]. TaqMan probes (Supplemental Table 2), qRT-PCR conditions and analysis, as well as RNAseq and global splicing analysis are described in the supplement.

### Statistics

Data are expressed as mean ± SEM. Multiple group comparisons were analyzed by two-way ANOVA. *P* values ≤ 0.05 were considered statistically significant.

### Accession codes

All mouse RNA sequencing data have been submitted to the NCBI sequence read archive (SRP091317).

## Results

### Reduced RBM20 expression restores cardiac dimensions in titin N2B-deficient mice

**Fig. 1:**
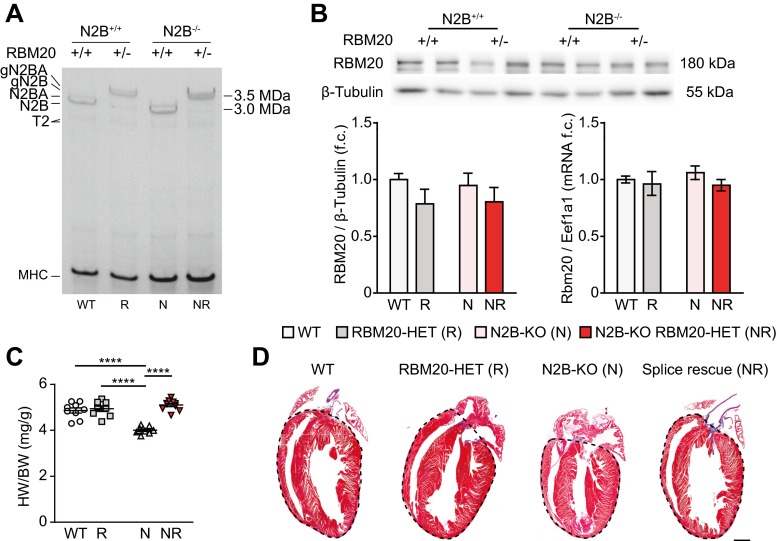
Reduced RBM20-dependent alternative splicing restores cardiac dimensions in titin N2B knockout hearts. **a** Titin isoform expression of left ventricles from wildtype (*WT*) vs. heterozygous RBM20-deficient (*R*) and N2B knockout (*N*) vs. compound RBM20/N2B-deficient animals (*NR*). **b** Protein and RNA expression analysis of RBM20. Representative western blot of RBM20 and the quantification normalized to β-tubulin in WT, RBM20-HET, N2B-KO, and splice-rescue mice (*n* = 6 for each group). Quantification of Rbm20 mRNA expression normalized to Eef1a1 (*n* = 5–6 for each group). **c** Heart weight (*HW*) to body weight (*BW*) ratios (*n* = 8–10 for each group). **d** Representative longitudinal images of Masson’s trichrome-stained hearts with ventricular area indicated by the *dashed line*. *Scale bar*, 1 mm; original magnification, ×5. *P < 0.05; **P < 0.01; ***P < 0.001; ****P < 0.0001 (**b**, **c**)

We bred the N2B-KO as an animal model with diastolic dysfunction [5] with the splice-deficient RBM20 knockout mouse lacking the RNA-binding domain (RBM20^∆RRM^). This mutation inefficiently removes titin I-band exons from the mature transcript and therefore increases the length of titin’s spring elements [11]. The resulting strain carries the homozygous N2B deletion (N2B-KO) and the heterozygous deletion of the RBM20 RRM domain (RBM20-HET). The animals display normal pre- and postnatal development, fertility, and weight gain (Supplemental Fig. S1a, b). We refer to these animals as “splice-rescue” mice (N2B-KO RBM20-HET; Fig. 1a). The N2B-deficient giant titin isoforms are expressed in similar amounts as the giant isoforms in RBM20-HET and run at the expected sizes in the titin gel. Loss of RBM20’s RRM does not lead to compensatory upregulation of RBM20, as RNA and protein expression over all RBM20 isoforms and truncations were unchanged in RBM20-HET and splice-rescue mice compared with the strains expressing wildtype RBM20 (Fig. 1b).

The heart-to-body-weight ratio of the N2B-KO is reduced [5], while heterozygous or homozygous deletion of the RBM20-RRM domain does not alter cardiac size [11]. Introduction of the RBM20^∆RRM^ allele into the N2B-KO reverts cardiac atrophy in splice-rescue animals: The heart-to-body-weight ratio is similar to wildtype levels (Fig. 1c) and ventricular geometry is restored as determined by trichrome staining of longitudinal cardiac slices (Fig. 1d). Neither the histology nor the real-time PCR for collagen isoform 1a2 (Supplemental Fig. S1c) provides evidence for ventricular fibrosis, which was reported in rats with a heterozygous deletion of RBM20 [2]. Trophic changes in the heart are usually accompanied by the upregulation of hypertrophy markers such as atrial natriuretic peptide (ANP) and brain natriuretic peptide (BNP). In N2B-KO mice, ANP and BNP messenger RNA (mRNA) levels are similar to WT mice, but both are significantly elevated in left ventricles of RBM20-HET and even more in splice-rescue mice compared with WT (Supplemental Fig. S1c). Myofiber thickness was not significantly different between genotypes (Supplemental Fig. S1d).

### Diastolic function is improved in splice-rescue mice

**Fig. 2:**
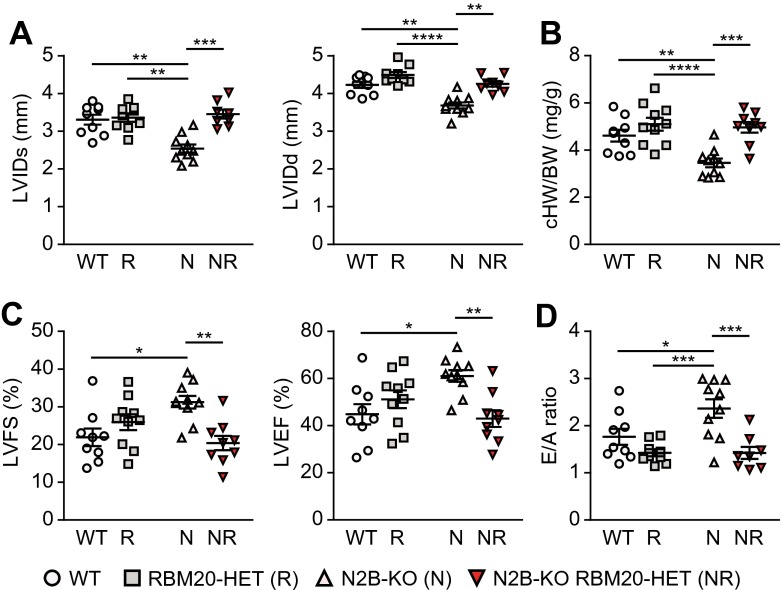
Heterozygous loss of RBM20 normalizes cardiac function in titin N2B-KO mice. Data in (**a–c**) were obtained by echocardiography and (**d**) by Doppler echocardiography. **a** Left ventricular inner diameter in diastole (*LVIDd*) and systole (*LVIDs*) in the four experimental groups (*n* = 9–10 for each group). **b** Calculated heart weight (*HW*) to body weight (*BW*) ratios (*n* = 9–10 for each group). **c** Left ventricular fractional shortening (*LVFS*) and left ventricular ejection fraction (*LVEF*) between genotypes (*n* = 9–10 for each group). **d** Mitral valve E-wave to A-wave ratio as a parameter of diastolic stiffness (*n* = 8–10 for each group). *P < 0.05; **P < 0.01; ***P < 0.001 ; ****P < 0.0001

The adaptation of cardiac size was validated by echocardiography with increased left ventricular inner diameter in systole and diastole of splice-rescue compared with N2B-KO mice (Fig. 2a). The calculated and measured heart-to-body-weight ratios were consistent across genotypes with reduced cardiac size only in the N2B-KO (Figs. 1c and 2b). Fractional shortening and ejection fraction were increased only in N2B-KO mice, with no significant difference between WT and splice rescue (Fig. 2c). To evaluate diastolic function, we used Doppler imaging of mitral inflow and found an elevated E/A ratio of N2B-KO mice, consistent with increased ventricular stiffness that results in less efficient filling (Fig. 2d). The E/A-ratio is reverted to WT levels in splice-rescue mice indicating normalized diastolic function. Other parameters of cardiac function were largely unchanged between genotypes (Supplemental Table 3).

**Fig. 3:**
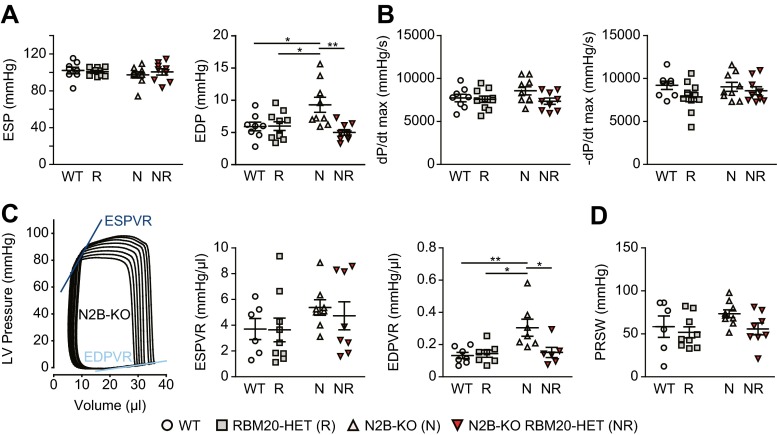
Reduced RBM20 activity decreases diastolic stiffness in N2B-KO hearts. Data in (**a–d**) were obtained by cardiac catheterization. **a** End-systolic (*ESP*) and end-diastolic (*EDP*) pressures in the four experimental groups (*n* = 8–10 for each group). **b** Changes in maximum velocity of contraction (dP/dt max) and maximum velocity of relaxation (−dP/dt max) between genotypes (*n* = 8–10 for each group). **c** Representative PV loops of the N2B-KO indicating end-systolic (*ESPVR*, *dark blue*) and end-diastolic pressure-volume relationships (*EDPVR*, *light blue*) and the quantification of ESPVR as a measure of end-systolic elastance and the EDPVR as a measure of diastolic stiffness (*n* = 6–7 for each group). **d** Preload recruitable stroke work (*PRSW*) as a measure of myocardial contractility (*n* = 6–9 for each group). *P < 0.05; **P < 0.01

As an independent method to evaluate diastolic function, we used conductance catheter-derived pressure volume relations to quantify left ventricular mechanics. End-systolic pressure was not changed between genotypes, but end-diastolic pressure was significantly increased in the N2B-KO and restored to wildtype levels in splice-rescue mice (Fig. 3a). End-systolic and end-diastolic volumes were decreased in the N2B-KO and increased in RBM20-HET, although not significantly (Supplement Table 4). Unlike maximum systolic pressure, maximum velocity of contraction (dP/dt), and maximum velocity of relaxation (−dP/dt), which were unchanged between groups (Fig. 3b; Supplemental Table 4), the end-diastolic pressure-volume relationship (EDPVR) of N2B-KO was significantly increased compared with all other groups (Fig. 3c). The reduced function of RBM20 restores EDPVR in splice-rescue mice to WT levels indicating normalized diastolic filling of the heart (Fig. 3c). The end-systolic pressure-volume relationship (ESPVR) and the preload recruitable stroke work (PRSW) were unchanged between groups (Fig. 3c). Combined, this data confirms the echocardiography findings of reverted diastolic dysfunction (Fig. 2d).

### Splice-rescue mice exhibit molecular adaptations to improve energy metabolism

**Fig. 4:**
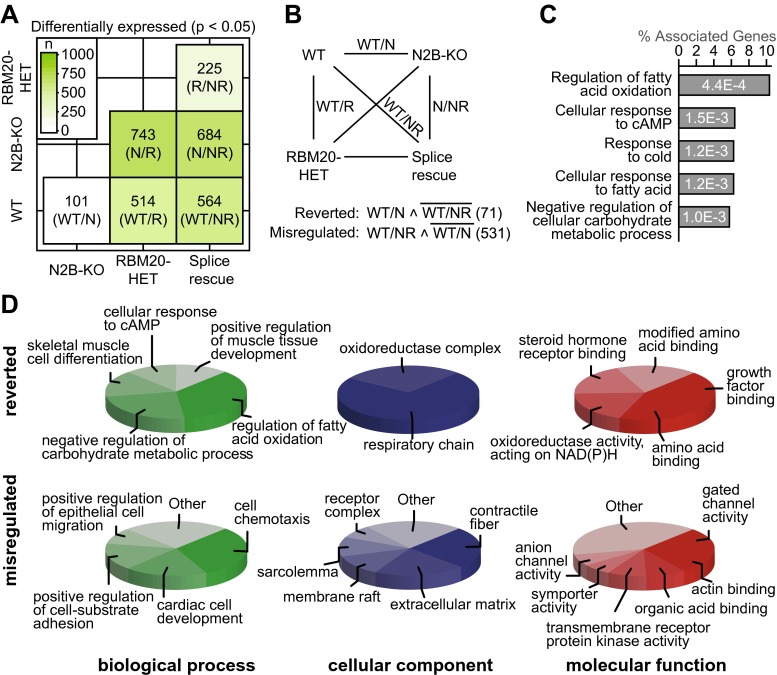
Whole transcriptome analysis by RNAseq. **a** Matrix of differentially regulated genes between WT, RBM20-HET, N2B-KO, and splice-rescue mice (*P* < 0.05; *n* = 2 for N2B-KO, *n* = 3 for other groups). Each square provides the number of differentially expressed genes per comparison. Color intensity reflects the gene count as indicated by the heat map. **b** Of the six possible comparisons between experimental groups, we focused differences of wildtype to the three mutants (WT/N, WT/R, WT/NR) and the comparison of N2B-KO with the splice rescue (*N/NR*). Genes differentially expressed between WT and N2B-KO but not between WT and splice rescue were normalized by reduced RBM20 activity (reverted genes). Genes differentially expressed between WT and splice rescue but not between WT and N2B-KO were additionally regulated by reduced RBM20 levels (misregulated genes). **c** Enrichment analysis of reverted genes (top 5 biological processes ranked by *P* value). **d** The reverted genes and misregulated genes were classified by their gene ontology

To evaluate the regulation of gene expression associated with the trophic changes in the splice rescue, we used high-throughput sequencing. The major transcriptional effect results from the heterozygous RBM20 deficiency, as pairwise comparisons between strains where only one group is RBM20 deficient produces 514 vs. 743 significantly deregulated transcripts. The knockout of titin’s N2B region accounts for 101 differentially regulated transcripts on the WT background and 225 transcripts on the RBM20-deficient background (Fig. 4a). To differentiate the benefit and adverse effects of introducing RBM20 deficiency in the N2B-KO on the molecular level, we identified transcripts with misregulated and transcripts with reverted expression after introduction of the Rbm20-deficient allele (Fig. 4b). The mutated Rbm20 allele caused an additional deregulation of 531 genes, which were not affected in the N2B-KO compared with WT (misregulated). Among the 101 genes differentially regulated between N2B-KO and WT, 71 genes were no longer differentially expressed in the splice-rescue animals (reverted). These 71 genes relate to the regulation of fatty acid and carbohydrate metabolism, as well as the cellular response to cAMP as determined by pathway enrichment analysis (gene ontology–biological processes; Fig. 4c). Reverted and misregulated genes were classified according to the gene ontology biological process terms, cellular component, and molecular function using Cytoscape (Fig. 4d). Genes with restored expression in the splice-rescue animals (reverted) relate to muscle development and metabolism (Fig. 4d). The gene products are enriched for localization in the respiratory chain (Fig. 4d; Supplemental Fig. S2) and relate to growth factor and amino acid binding or oxidoreductase activity. The misregulated genes are involved in cardiac development, migration, and cell adhesion indicating a role in remodeling. The gene products are located in the extracellular matrix and contractile fibers (binding actin) and relate to ion transport and actin binding. Together the expression changes between the N2B-KO with diastolic dysfunction and the splice-rescue animal suggest normalization of cardiac metabolism and altered remodeling.

### Decreased functional RBM20 protein shifts cardiac isoform expression

**Fig. 5:**
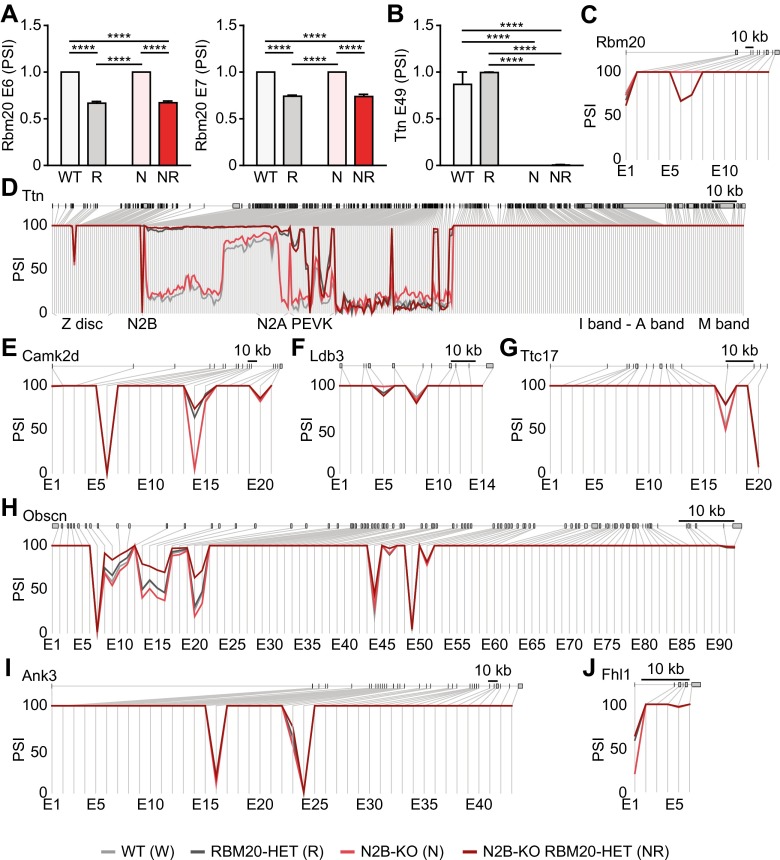
RBM20-dependent isoform expression. Differential inclusion of RBM20 exons 6 and 7 (**a**), titin N2B region (**b**), and genotype-dependent isoform expression of full-length Rbm20 (**c**), Ttn (**d**). genotype-dependent isoform expression of Camk2d (**e**), Ldb3 (**f**), Ttc17 (**g**), Obscn (**h**), Ank3 (**i**), and Fhl1 (**j**). PSI scores (percentage spliced in) are indicated on the *y*-axis for each experimental group (*n* = 2 for N2B-KO, *n* = 3 for other groups). Exon labels are below; size bar, 10 kb. ****P < 0.0001

To identify the murine splice targets of RBM20 and evaluate their contribution to improving the cardiac phenotype of N2B-KO mice, we analyzed the differential isoform expression between WT, RBM20-HET, N2B-KO, and splice-rescue mice. The exon level analyses independently validated the genotyping results and the heterozygous deletion of exons 6 and 7 of Rbm20 in RBM20-HET and splice-rescue mice (Fig. 5a, c) and the homozygous deletion of the N2B element in N2B-KO and splice-rescue mice (Fig. 5b, d). In both, the RBM20 heterozygous and N2B-KO animals, we find partial compensation at the knockout locus: heterozygous deletion of Rbm20 does not lead to the expected 50 % reduction in Rbm20 exons 6 and 7, suggesting upregulation of the WT vs. KO allele on the transcript level (Fig. 5a, c). Along the titin elastic region, additional exons encoding PEVK and IG domains are included in the final N2B-deficient transcript as PSI values of these exons are increased in N2B-KO vs. WT (Fig. 5d). In N2B-KO mice, changes in isoform expression are largely confined to titin itself, as PSI values in other genes are unchanged. The heterozygous mutation of Rbm20 in RBM20-HET and splice-rescue mice leads to several changes in isoform expression as compared with WT and N2B-KO mice. In total, 106 genes were differentially affected on the exon level (FDR <0.01). These potential substrates of murine RBM20 relate to myofibril assembly, respiratory chain, the ribosome, and the muscle myosin complex as determined by gene ontology analysis (Supplemental Fig. S3). Thirty-nine genes were consistently spliced between RBM20-HET and splice rescue vs. WT, and five of them have previously been identified as RBM20 binding transcripts [14].

The main target of RBM20-dependent splicing in the mouse is titin. The heterozygous deletion of RBM20’s RRM leads to an up to 4-fold increase in PSI values of alternative exons in titin’s elastic region located between the N2B element and the PEVK region (Fig. 5d). Several transcripts are differentially spliced by RBM20, with exon skipping or alternative exon inclusion in Camk2d, Ldb3, Ttc17, Obscn, and Ank3 (Fig. 5e–i). Camk2d and Ldb3 have been described as RBM20 targets in the Rbm20-deficient rat [2, 14] and obscurin—although not significantly regulated in the rat—has been identified based on the physical interaction of the transcript with RBM20 [14]. The newly identified RBM20-dependent isoforms expressed from the Ttc17, Ank3, Fhl1, Arhgap10, Cflar, Hmgb1, and Myh7 gene (Fig. 5; Supplemental Fig. S4 and S6) relate to actin polymerization, sarcomere structure and mechanotransduction. A summary of the gene functions and exons/domains affected is provided in Supplemental Table 5. Importantly, we find one differentially expressed isoform of the titin binding protein FHL1, with differential inclusion of the first exon that includes the ATG (Fig. 5j). RBM20 deficiency would increase inclusion of the exon containing the translation start and thus increase FHL1 protein expression.

### FHL1 expression is increased in splice-rescue hearts

**Fig. 6:**
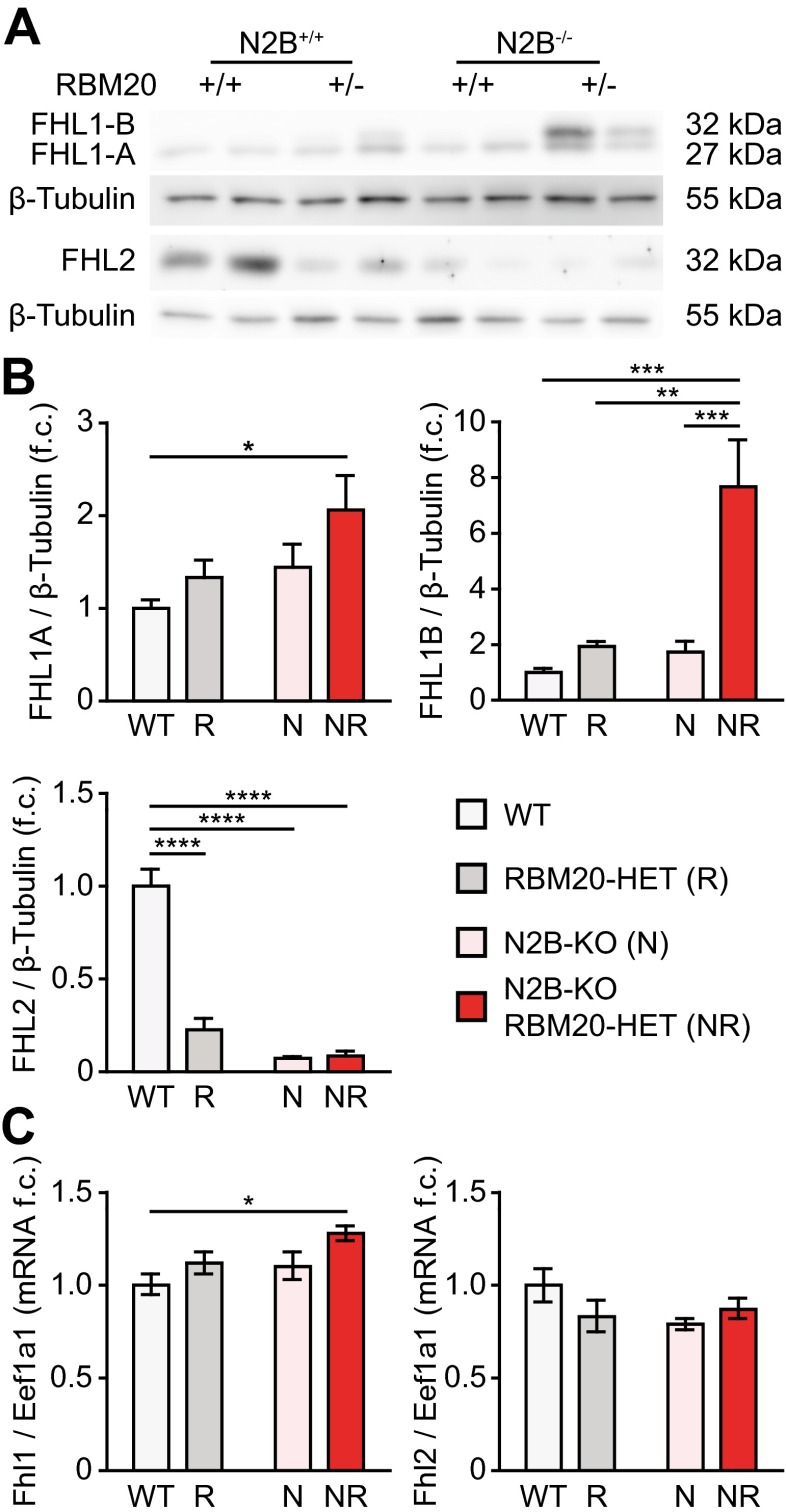
FHL1 and FHL2 levels are inversely regulated in splice-rescue mice. **a** Representative western blot analysis of FHL1 and FHL2 expression levels in LV tissue of WT, RBM20-HET (*R*), N2B-KO (*N*), and splice-rescue mice (*NR*). **b** Quantification of FHL1A, FHL1B, and FHL2 protein expression normalized to β-tubulin (*n* = 6 for all groups). (**c**) Quantification of Fhl1 and Fhl2 mRNA levels by TaqMan normalized to Eef1a1 (*n* = 5–6) *P < 0.05; **P < 0.01; ***P < 0.001; ****P < 0.0001

To address the molecular basis of the restored cardiac dimensions of splice-rescue mice, we analyzed the expression of several proteins previously linked to cardiac hypertrophy signaling such as Erk1/2, Akt, mTOR, NFATc1, and JNK. All were unchanged between genotypes (Supplemental Fig. S5a–e). In addition, we measured protein levels of the four-and-a-half lim-domain proteins FHL1 to determine, if the additional inclusion of the RBM20-dependent exon containing the ATG would increase FHL1 levels vs. FHL2, which is not differentially spliced by RBM20 (Fig. 6a, Supplemental Fig. S4e). Both FHL1 and FHL2 have not only been linked to cardiac hypertrophy signaling but also bind titin [15, 16]. FHL1 protein levels—predominantly the FHL1B isoform—are strongly increased only in splice-rescue to WT (Fig. 6a, b). Left ventricular mRNA levels are largely unchanged with minor upregulation of Fhl1 RNA in splice-rescue compared with WT mice, suggesting posttranscriptional regulation (Fig. 6c). Thus, the reversion of diastolic dysfunction and cardiac atrophy in N2B-KO mice by reduced expression of functional RBM20 is dependent on titin’s mechanical properties and associated with a posttranscriptional effect on FHL1, which links titin-based biomechanics to cardiac hypertrophy signaling.

## Discussion

Diastolic dysfunction is an important contributor to the pathophysiology of heart failure, and our recent identification of RBM20 as a titin splice factor has provided a unique target to adjust the diastolic properties of the heart [2]. We chose a genetic approach to adjust alternative splicing and concertedly affect multiple protein isoforms that adapt cardiac function in the titin N2B-KO mouse. In this animal model, the loss of titin’s heart-specific elastic N2B region leads to a complex cardiac phenotype including diastolic dysfunction [5]. Combining the N2B mutant with a splice-directed therapeutic approach allowed us to address the following questions: first, can increased titin-based stiffness resulting from loss of one elastic domain be compensated by the inclusion of another structural region? Second, can synergistic adaptation of multiple mRNA isoforms restore a complex cardiac phenotype?

In the splice-rescue mouse, the diastolic dysfunction resulting from the loss of the titin N2B domain is completely restored, as determined by echocardiography and conductance catheter analysis. It includes the reduced E/A ratio as a sign of improved ventricular filling, normalized passive pressures, and normalized pressure volume relations. These findings not only suggest that adjusting exon inclusion via inhibition of RBM20 can improve the biomechanical properties of the heart. But also, they indicate that restoration of compliance does not have to occur at the protein-domain that caused the increase in stiffness, as the adjustment of PEVK and IG domain-based elasticity can compensate for the altered N2B-based stiffness. Furthermore, our data also indicate that even a reduction of RBM20 function to only 50 % is sufficient to restore diastolic function, increasing the therapeutic window of an RBM20-directed therapy. These findings are significant in light of the series of unsuccessful attempts to significantly improve cardiac function in patients with diastolic dysfunction [17–20]. Finally, the reduced RBM20 activity affects the whole organism but does not lead to an obvious extracardiac phenotype (weight, fertility, grooming), indicating a sufficiently large therapeutic window for the future development of an RBM20 inhibitor.

In N2B-KO animals, reduced RBM20 activity not only improved diastolic dysfunction but also reverted the cardiac atrophy of titin N2B-deficient diastolic dysfunction model, illustrated by heart-to-body-weight measurements, trichrome stainings, echocardiography and hemodynamic catheter analysis. On the molecular level, we found a positive effect on metabolic function as reflected in reverted expression of genes related to fatty acid oxidation. This improvement could result from a direct effect of the differential splicing of RBM20 substrates that relate to cardiac hypertrophy signaling. As a secondary effect, improved diastolic function might change ventricular geometry as filling is improved or reduce strain of the ventricular wall that might lead to improved coronary flow and thus increased availability of nutrients. On a molecular level, altered titin-based elasticity could change localization or stability of proteins that differentially bind to the extended vs. relaxed titin filament [6].

We used RNAseq analysis to evaluate which transcripts are alternatively spliced dependent on RBM20. At the exon level, we find several genes affected, with more than 50 potential novel substrates of RBM20. These genes relate to the regulation of relaxation of cardiac muscle and myofibril assembly, which could explain part of the beneficial effect on the phenotype. Several genes are relevant to sarcomere structure and the hypertrophy response. In addition to candidate genes with relation to the improved cardiac phenotype, we thus identified several RBM20 substrates, including Obscurin, which was not significantly affected on the exon level in the RBM20-deficient rat [2] but has been identified by the CLIP analysis [14]. For select transcripts, we found differential exon inclusion between RBM20-deficient and splice-rescue animals, which indicates a feedback of increased titin-based stiffness in the N2B-KO on alternative splicing and a potential primary role in the restored cardiac function of splice-rescue animals. Finally, the first exon level analysis of the titin N2B knockout revealed an unexpected finding with increased inclusion of I-band exons that would partially revert the increased stiffness resulting from the loss of the N2B exon. As RBM20 protein levels are not different between N2B knockout and wildtype animals, this effect could be mediated at the posttranslational level or via an RBM20-independent splice factor.

Secondary changes reflected in our analysis of the RNAseq data at the gene level can indicate adverse effects of targeting RBM20 or beneficial effects. Genes that are reverted, namely deregulated in N2B-KO but no longer in the splice-rescue animals, are indicative of a compensatory regulation. They relate to the regulation of fatty acid oxidation and the cellular response to cAMP. The reversion of genes related to beta oxidation affects all steps of the electron transfer chain, suggesting a concerted regulation resulting in improved energy balance in the splice-rescue animals. Thus, positive effects of a splice level therapeutic approach via RBM20 could synergistically improve diastolic function, size and metabolism.

To investigate the molecular basis of the trophic phenotype, we investigated the regulation of several hypertrophy pathways and their contribution to the restored cardiac size of the splice-rescue animals. We have previously proposed that the decreased trophic signaling in the N2B-KO heart is caused by decreased protein levels of the titin N2B-binding protein FHL2 [5]. Interestingly, not only the deletion of the N2B region led to a decrease in FHL2 protein levels but also the heterozygous deletion of RBM20’s RRM—although the effects were not additive (splice-rescue mice expressed similar amounts of FHL2 as the N2B-KO). Thus, altered FHL2 protein levels are not sufficient to explain the increased cardiac trophic signaling in splice-rescue mice. In addition to the decreased FHL2 protein level, we found a minor increase of the titin binding protein FHL1 and a highly significant increase of its alternative splice isoform FHL1B in splice-rescue mice (~7.5-fold increase compared with WT). FHL1 mRNA levels were only mildly upregulated in splice-rescue mice and the FHL1 isoform that differentially includes the ATG-exon to improve translation only accounts for <10 % of the total FHL1 mRNA. Thus, the increase in FHL1 protein levels occurs at a posttranscriptional level and would be consistent with increased translation or decreased degradation of FHL1. FHL1 binds to titin’s N2B region, where it has been suggested to form a complex, which induces hypertrophy via Raf, MEK1/2 and ERK2 [15, 21]. Here, we do not find significant changes in ERK2 activity. Thus, the isolated increase in FHL1 protein levels in the absence of titin’s N2B region links FHL1 to mechanically induced trophic signaling that is independent on the N2B region as a force sensor and the induction of Erk phosphorylation. How the combination altered titin-based wall stress and deregulation of FHL1 can increase trophic signaling to restore cardiac size will have to be resolved in future experiments.

**Fig. 7:**
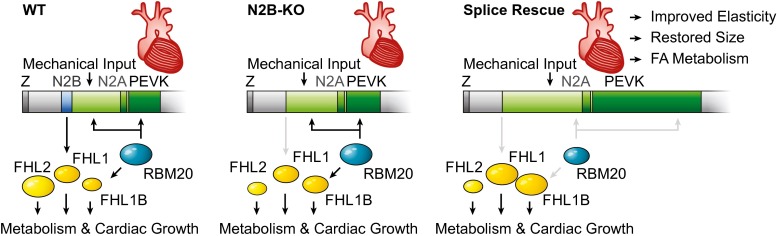
Splice-directed therapy in N2B KO mice with diastolic dysfunction and cardiac atrophy. Loss of the elastic N2B region (*blue*) leads to cardiac atrophy with reduced FHL2 and increased FHL1B expression. Reducing RBM20 activity to 50 % (splice rescue) increases exon retention both in titin’s I-band region (*green*) and in FHL1B. Together the RBM20-dependent adaptation of cardiac splicing restores titin-based elasticity and cardiac size and adapts FHL protein levels and fatty acid (FA) metabolism

In summary, we extended the RBM20 substrate spectrum in the mouse and found several alternatively spliced genes that relate to sarcomere structure and mechanosignaling. Reduced expression of functional RBM20 in the N2B-KO leads to a compensatory inclusion of titin I-band exons into the N2B-deficient titin resulting in decreased titin-based stiffness. Increased inclusion of the FHL1 exon containing the translational start in RBM20-deficient mice leads to the expected increase in FHL1 protein levels (Figs. 6 and 7). Together, these changes would not only improve the mechanical properties of the sarcomere but also should facilitate mechanically induced hypertrophy signaling. Reduced RBM20 activity reverts both diastolic dysfunction as well as cardiac atrophy and partially normalizes the expression of genes that relate to striated muscle development and fatty acid metabolism. The validation of RBM20 as a therapeutic target in heart failure, combined with our published RBM20 splice reporter assay [2], could provide a suitable basis for the future development of splice inhibitors to improve diastolic function.

## Electronic supplementary material

ESM 1 (DOCX 1.25 mb)
