# Supplementary material for: Reducing RBM20 activity improves diastolic dysfunction and cardiac atrophy
Source: J Mol Med (Berl). 2016 Nov 26;94(12):1349–58. doi: 10.1007/s00109-016-1483-3 (PMC5143357; doi:10.1007/s00109-016-1483-3)
Supplement: Supplementary file 1 — (DOCX 1.25 mb) [file 109_2016_1483_MOESM1_ESM.docx]

# J Mol Med 2016

# Electronic Supplemental Material

Reducing RBM20 activity improves diastolic dysfunction and cardiac atrophy

Florian Hinze^1,4^, Christoph Dieterich^2,5^, Michael H Radke^1,4^, Henk Granzier^3^ & Michael Gotthardt^1,4^

1. Neuromuscular and Cardiovascular Cell Biology, Max-Delbrück-Center for Molecular Medicine, 13125 Berlin, Germany
2. Klaus Tschira Institute for Integrative Computational Cardiology and Dept. Cardiology, Angiology, and Pneumology, Heidelberg University, 69120 Heidelberg, Germany
3. Depts. of Physiology and Cellular and Molecular Medicine, University of Arizona, Tucson, AZ
4. DZHK (German Centre for Cardiovascular Research), partner site Berlin, Berlin, Germany
5. DZHK (German Centre for Cardiovascular Research), partner site Heidelberg, Heidelberg, Germany

# Supplemental Experimental Procedures

### Animal procedures

The titin N2B knockout as a model for diastolic dysfunction, as well as the RNA recognition motif knockout of RBM20 have previously been described [1, 2]. Male mice were sacrificed by cervical dislocation at 100 to 120 days of age. The hearts were rapidly excised, washed in PBS, and dissected into atria, septum, right and left ventricle. The tissue were snap frozen in liquid nitrogen and stored at -80°C. For further analyses left ventricular tissue was processed to tissue powder using liquid nitrogen cooled mortars and pestles. We evaluated the cardiac dimensions and functions by echocardiography and hemodynamic catheter analysis. For genotyping we used DNA extracted from ear tags. All experiments involving animals were carried out following institutional and US National Institutes of Health guidelines as approved by LaGeSo Berlin. Mice were age- and sex-matched for analysis.

### Generation and genotyping of the splice rescue strain

The N2B splice rescue mice were generated by crossing the Ttn exon 49 (N2B exon) deletion model [1] and the Rbm20 exon 6 and 7 (RNA recognition motif) deletion model [2]. Both exonic deletions are in-frame. N2B splice rescue mice were maintained on a 129S6 background (Taconic). For genotyping, DNA was extracted from murine ear tags. The deletions of the N2B exon and Rbm20 exon 6 and 7 were confirmed by PCR. The N2B deletion were detected with N2B_f (5’- AATCTCACCACAACCTTATTCCA -3’) and N2B-WT_r (5’- GGTTAACAGCATCCCATTAAAGA -3) for the wild type allele and N2B-KO_r (5’- AGTGAATTGCGGGGAAATTATTA -3’) for the knockout allele. The exon 6 and 7 deletion of Rbm20 were detected using Rbm20_f (5’- ATATCTGCACCCATGTTTAGTTTCC -3’) and Rbm20-WT_r (5’- GAAGCCAGTGTGTTGGTATGG -3’) for the wild type allele and Rbm20-KO_r (5’- ATTTGAATGCCCCCAGAAGT -3’) for the knockout allele.

### Analysis and quantification of protein expression

Proteins were extracted from left ventricular tissue powder. Tissue powder was incubated with RIPA buffer (50 mM Tris pH 8.0, 150 mM NaCl, 0.25% Na-deoxycholate, 1% NP-40, 1 mM EDTA, 1 mM EGTA) for 30 min on ice followed by sonication. Then samples were centrifuged at 13000 rpm for 20 min, flash frozen in liquid nitrogen and stored at -80°C. The proteins were separated on a SDS-PAGE gel system (Biorad) and transferred to Hybond P PVDF membranes (GE Healthcare) for 2 h with 200 mA for subsequent western blot analysis. Blocking were performed in superblotto (10 mM Tris-HCl pH 8.0, 150 mM NaCl, 0.1 % Tween 20, 0.5% BSA, 2.5 % milk powder) for 1 h at room temperature followed by the incubation with primary antibody overnight at 4°C. Antibodies were used according to manufacturer’s instructions and are listed in Supplemental Table 1. The detection was performed using horseradish peroxidase-conjugated secondary antibodies and chemiluminescence staining using ECL (Supersignal West Pico/Femto Chemiluminescence Substrate, Pierce) on a Fusion Fx7 system (PeqLab). The software AIDA v4.23 was used for quantification. Analysis of titin isoform expression was performed as described previously [3]. Tissue powder was homogenized in 40x volume sample buffer (8 M urea, 2 M thiourea, 3% SDS, 0.03% bromophenol blue, 75 mM DTT, 0.05 M Tris pH 6.8) by douncing for 30 s at 60°C. Then glycerol was added to a final concentration of 12% and the tissues were homogenized for 2 more min at room temperature, aliquoted and snap frozen in liquid nitrogen. The homogenized samples were electrophoresed on a 1% agarose gel using vertical SDS-agarose gel system (Hoefer). The gels were run at 15 mM for 4 h followed by Coomassie brilliant blue staining to visualize titin isoforms N2BA, N2B, and the proteolytic fragment T2 and MHC.

### Trichrome staining

Mice at 100 to 120 days of age were weighed and sacrificed by cervical dislocation. The hearts were rapidly excised, washed in PBS and fixed with 4% PFA overnight at 4°C. The hearts were rehydrated in H_2_O, subsequently dehydrated in an ascending ethanol series, embedded in paraffin, sectioned and a trichrome staining were performed. Stained sections were then imaged using a DMI 6000 B microscope (Leica, 5x magnification).

### Cardiac myofiber size determination

The diameters of cardiac myofibers were determined from 20x magnified trichrome stained slices using ImageJ (Fiji).

### RT-PCR and RT-qPCR

Total RNA was extracted from left ventricular tissue powder with TRIzol (Invitrogen) following the manufacturer’s protocol. At least five individual left ventricles were processed per genotype. A clean-up of the RNA was performed using RNAeasy mini spin columns (Qiagen). The RNA was reverse transcribed to cDNA using the high-capacity RNA-to-cDNA kit (Thermo Fisher Scientific). Quantitative RT-PCR was performed using TaqMan master mix (Applied Biosystems) in a 7900 HT cycler (Applied Biosystems). RT-PCR primers and TaqMan probes are listed in Supplemental Table 4. The quantification of the gene expression was performed using the ∆∆C_T_ method using Eef1a1 as the reference gene [4].

### Echocardiography

For echocardiography age and sex matched mice (100-120 days old males) were anesthetized with an intermixture of oxygen and 2.5% isoflurane. The fur was removed using a hair removal cream and a shaver. Contact gel was used for an optimal imaging of the transducer. Vital parameters as cardiac and breathing frequency were measured by electrodes at the paws. The ultrasonic probe MS-400 was used to determine the cardiac parameters and the VEVO 2100 system (Visualsonics Fujifilm) for visualization as described previously [5].

### Conductance catheter measurement

For the cardiac pressure and volume measurement the Millar Conductance Catheter System was used as described previously [5]. Age and sex matched mice (100-120 days old males) were anesthetized with an intermixture of oxygen and 2.5% isoflurane, orotracheally intubated and ventilated. The catheter was inserted retrogradely into the left ventricle via the arteria carotis communis to measure pressure volume relations. The artery was exposed by cervical incision between lower jaw and sternum. Blood was arrested proximal and distal of the artery. The placement of the catheter was validated by electrocardiography (Vevo2100). Pressure independent parameters were measured after a three second occlusion of the vena cava inferior. The vein was exposed by opening the peritoneum. The occlusion causes a pressure decrease and reduction of the left ventricular volume.

### RNAseq library prep and analysis of RNAseq data

RNA from three left ventricles was isolated using TRIzol followed by a clean-up with the QIAGEN RNA micro kit. The cDNA libraries were generated using the Illumina TruSeq Stranded mRNA Sample Prep Kit and 2x101 paired end sequenced on HiSeq2000. The reads were trimmed and quality clipped with Flexbar [6]. All remaining reads greater 18bp were mapped to the murine genome (assembly EnsEMBL 84) with the splice-aware STAR aligner [7]. Subsequent transcriptome analyses on differential gene and isoform abundance were carried out with cuffdiff [8]. The differentially expressed and differentially spliced genes (P < 0.05) were analyzed and clustered by their biological processes, molecular function and biological component Cytoscape v3.3.0 and the plug-in ClueGO v 2.2.5.

### Global splicing analysis

We tested for differential exon usage in pair-wise comparisons for RBM20-HET, N2B-KO and splice-rescue mice compared to WT to identify exons regulated by Rbm20. Percentage spliced in values were computed as conceptually outlined in Schäfer et al. [9]. However, we used the more recent StringTie software [10] to re-annotate the reference transcriptome and to compute splice junction read counts. Custom PERL and R scripts were employed to integrate the relevant junction and exonic read counts to arrive at a final list of PSI values.

# Supplemental Figures

**Figure S1**


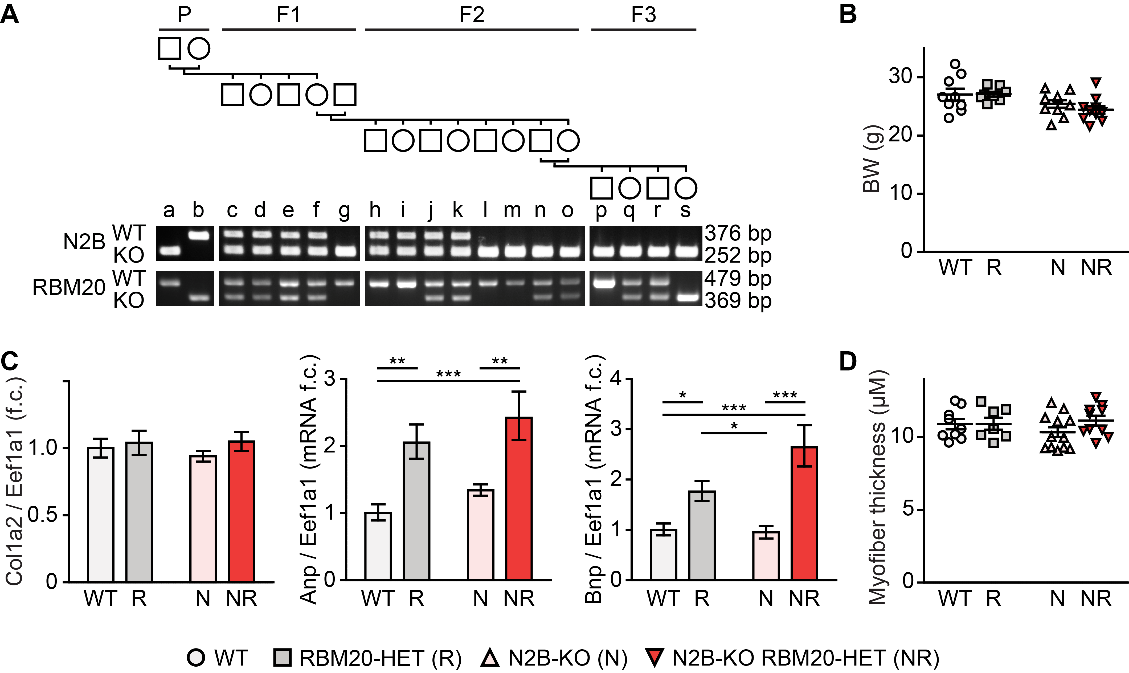


**Supplemental Figure 1: Generation and validation of N2B splice rescue model.** (**A**) Breeding scheme with genotypes. Genotyping PCRs for N2B and RBM20 generated a short fragment for knockout allele and a longer for the wildtype allele. (**B**) Body weight of each mouse strain. (**C**) Quantification of Col1a2, Anp, and Bnp mRNA levels by TaqMan normalized to Eef1a1 (n = 5 for WT; n = 6 for other groups). (**D**) Diameter of cardiac myofibers.


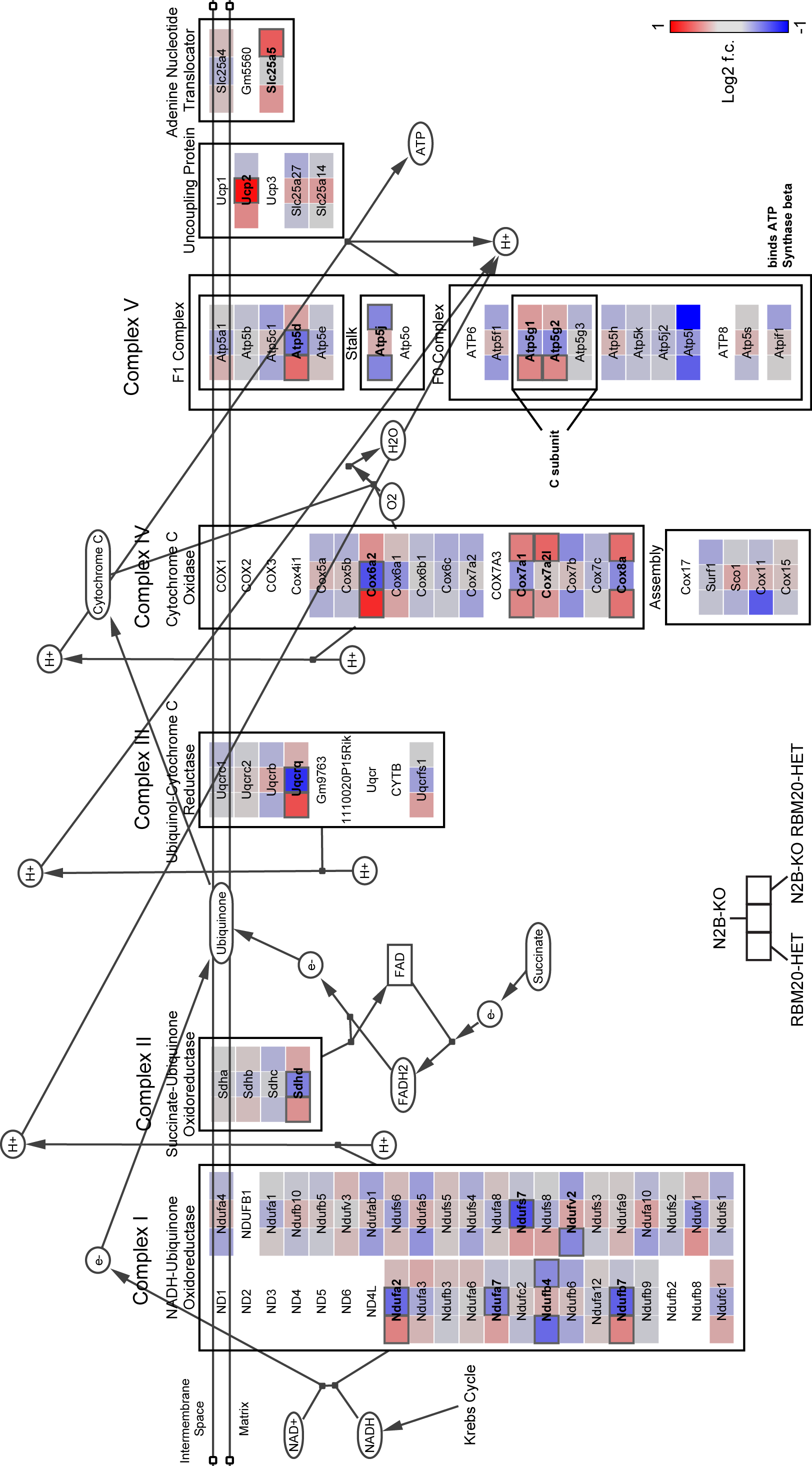
**Figure S2**

**Supplemental Figure 2: Restored expression of genes related to oxidative phosphorylation (RNAseq data overlaid on the WIKIpathway electron transport chain)**. The color code defines gene expression relations to WT mice in Log2 fold change ranging from 1 to -1 (red: upregulated, blue: downregulated). Each box representing a gene is split in three squares to indicate the regulation in RBM20-HET, N2B-KO and N2B splice rescue vs. WT (left to right). Genes significantly regulated vs. WT (P < 0.05) are framed in dark grey and bold.

**Figure S3**


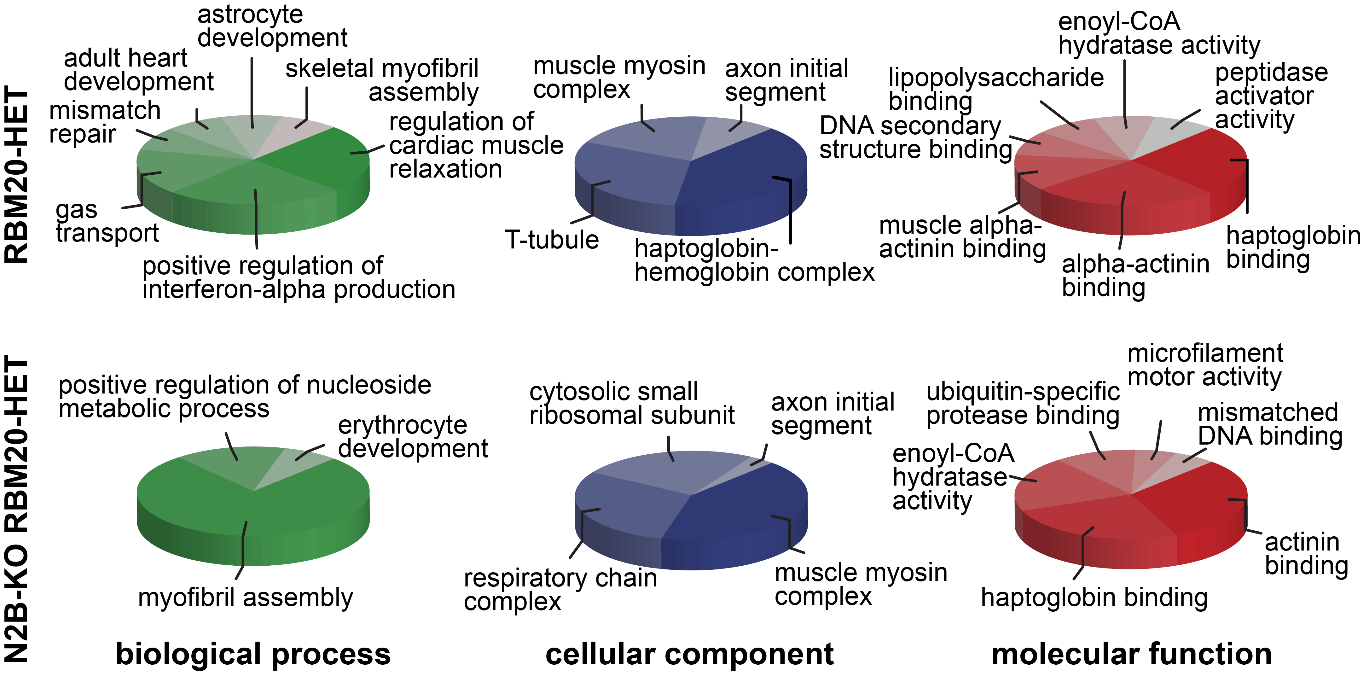


**Supplemental Figure 3: Gene ontology classification of differentially spliced genes.** Transcripts differentially spliced between RBM20-HET (top) and N2B splice rescue mice (bottom) as compared to WT mice were classified by gene ontology analysis (biological process, cellular component, and molecular function).

**Figure S4**

**
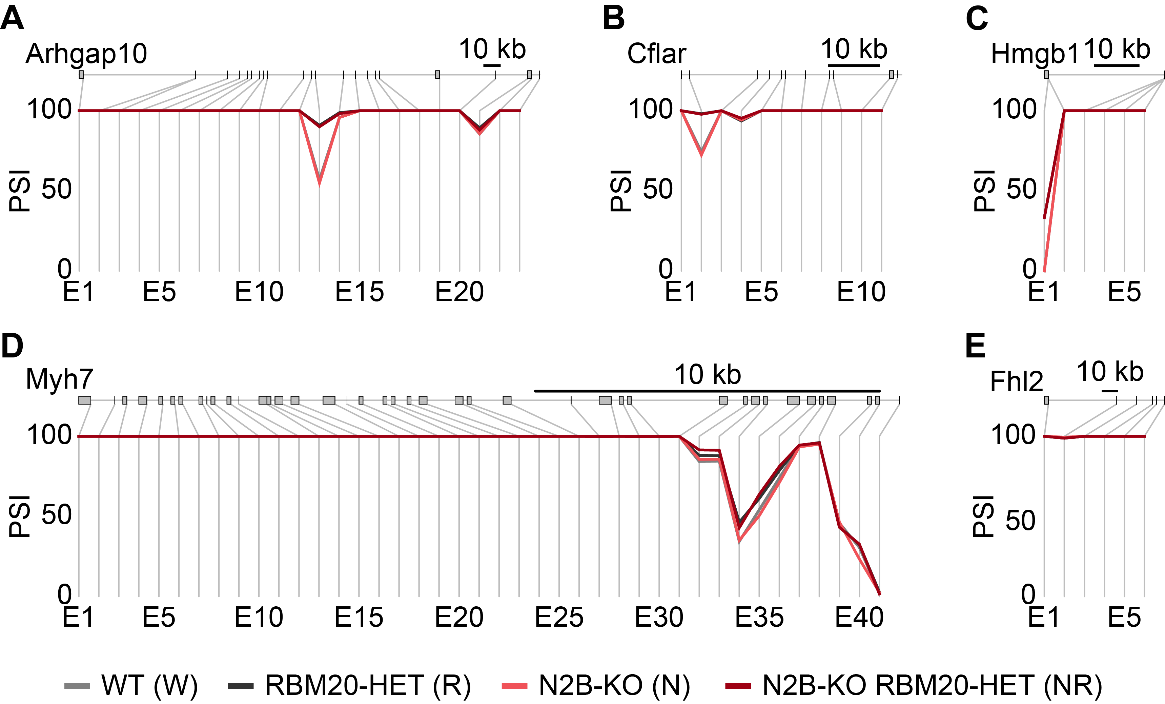
**

**Supplemental Figure 4: RBM20-dependent isoform expression (cont. from Fig. 5).** Genotype dependent isoform expression of Arhgap (**A**), Cflar (**B**), Hmgb1 (**C**), Myh7 (**D**), and Fhl2 (**E**). PSI scores (percentage spliced in) are indicated on the Y axis (n = 2 for N2B-KO, n = 3 for other groups). Exon labels are below; size bar (10 kb).

**Figure S5**


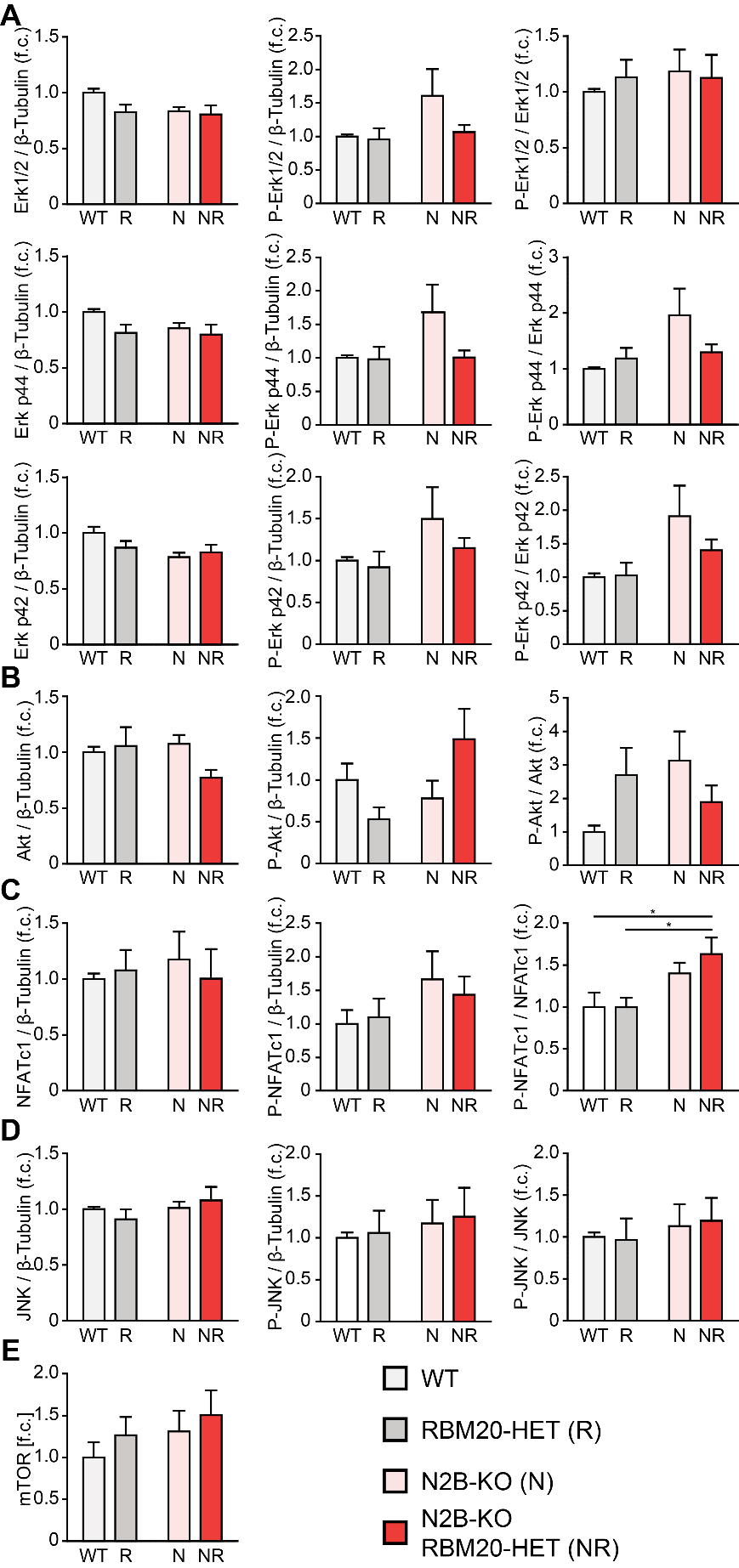


**Supplemental Figure 5: Total and phospho protein levels of Erk1/2 and Akt.** (**A**-**E**) Quantification of total- and phospho-protein normalized to beta-Tubulin, and the ratio of phospho to total protein levels of Erk1/2 (**A**), Akt (**B**), NFATc1 (**C**), JNK (**D**) and mTOR (**E**).

**Figure S6**


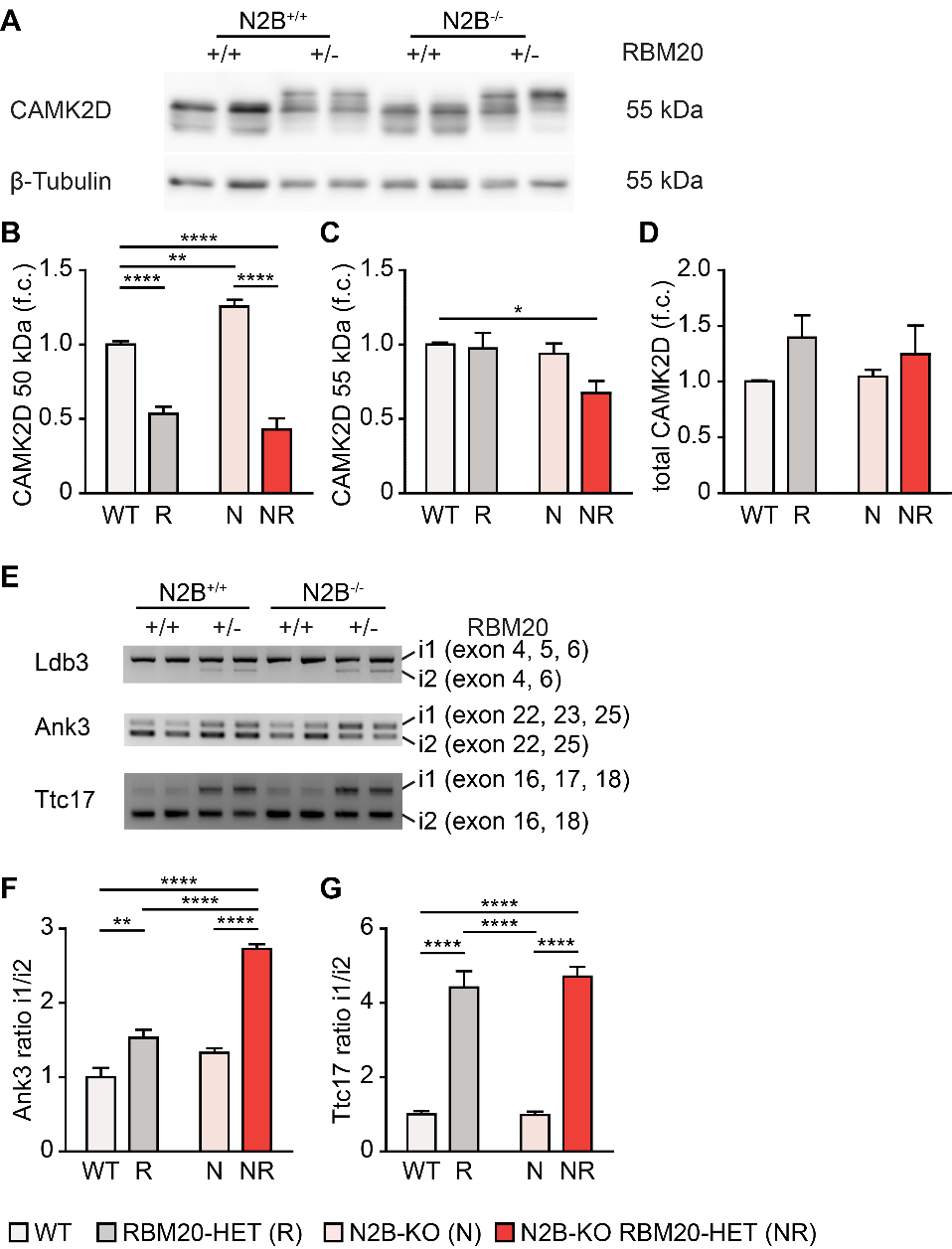


**Supplemental Figure 6: Validation of differential isoform expression.** (**A**) Genotype dependent isoform expression of Camk2d validated by western blot (n = 6 per group). (**B-D**) Quantification of Camk2d isoform expression normalized to WT and the house keeping gene β-tubulin. (**E**) Validation of RBM20-dependent isoform expression of Ank3, Ldb3, and Ttc17 by RT-PCR. (n = 4-6 per group). (**F, G**) Quantification of RBM20-dependent Ank3 and Ttc17 isoform expression.

# Supplemental Tables

## Supplemental Table 1: Antibodies

| **Antibodies** | **Order no** | **Supplier** |
| --- | --- | --- |
| Akt | 4691 | Cell Signaling |
| Phospho-Akt | 4060 | Cell Signaling |
| β-Tubulin | 2146 | Cell Signaling |
| CAMK2D | ab181052 | Abcam |
| Erk1/2 | 9102 | Cell Signaling |
| Phospho-Erk1/2 | Sc-7383 | Santa Cruz |
| FHL1 | 58067 | Abcam |
| FHL2 | K0055-3 | MBL |
| JNK | 9252 | Cell Signaling |
| P-JNK | 9255 | Cell Signaling |
| mTOR | 2983 | Cell Signaling |
| NFATc1 | sc-13033 | Santa Cruz |
| Phospho-NFATc1 | sc-32979 | Santa Cruz |
| RBM20 | custom | Eurogentec [11] |

## Supplemental Table 2: PCR primers and TaqMan probesets

| **Primer** | **Sequence** |
| --- | --- |
| Ank3_E22_f | CATCACGGAGAAGCACAAAA |
| Ank3_E25_r | GGCTCCAAGACTGAAGCCTA |
| Ldb3_E3_fw | CCTATTCCCATCTCCACGAC |
| Ldb3_E7_rev | GAGACTGCAGGTTGGAGGAC |
| Ttc17_E16_fw | CTTCAGACAGGCCTTGAAGC |
| Ttc17_E18_rev | CTCCACCTCGTCAGAACCAT |
| **Primer and Probes** | **Sequence/Applied Biosystems catalogue number** |
| ANP | Mm01255747_g1 |
| BNP fw | 5’-AGCTGCTGGAGCTGATAAGAGAA-3’ |
| BNP rev | 5’-GTGAGGCCTTGGTCCTTCAA-3’ |
| BNP probe | 6-FAM-AGTCAGAGGAAATGGCCCAGAGACAGCTA-TAMRA |
| Col1a2 | Mm01165187_m1 |
| EEF1a1 | Mm01973893_g1 |
| Fhl1 | Mm00515772_m1 |
| Fhl2 | Mm00515781_m1 |
| Rbm20 | Mm01264996_m1 |

## Supplemental Table 3: Echocardiography data

|  | **WT** | **RBM20-HET** | **N2B-KO** | **N2B splice rescue** |
| --- | --- | --- | --- | --- |
| **BW (g)** | 24.9 ± 0.9 | 26.3 ± 1.4 | 26.4 ± 0.6 | 23.5 ± 0.4 |
| **HR (bpm)** | 376 ± 22 | 432 ± 12 | 435 ± 8 | 432 ± 22 |
| **IVSd (mm)** | 0.73 ± 0.03 | 0.77 ± 0.03 | 0.7 ± 0.01 | 0.75 ± 0.03 |
| **IVSs (mm)** | 1.04 ± 0.05 | 1.06 ± 0.06 | 1 ± 0.02 | 1.02 ± 0.05 |
| **LVPWd (mm)** | 0.73 ± 0.02 | 0.78 ± 0.03 | 0.74 ± 0.02 | 0.76 ± 0.03 |
| **LVPWs (mm)** | 0.93 ± 0.03 | 1.04 ± 0.06 | 1.07 ± 0.05 | 0.98 ± 0.06 |
| **LVIDd (mm)** | 4.23 ± 0.08 | 4.41 ± 0.11 | 3.68 ± 0.08** ^####^ ^$$^ | 4.17 ± 0.11 |
| **LVIDs (mm)** | 3.3 ± 0.13 | 3.27 ± 0.13 | 2.54 ± 0.11** ^##^ ^$$$^ | 3.33 ± 0.16 |
| **LV FS (%)** | 21.9 ± 2.3 | 25.9 ± 2.1 | 31.2 ± 1.7* ^$$^ | 20.4 ± 1.9 |
| **LV EF (%)** | 44.8 ± 4.3 | 51.1 ± 3.7 | 61 ± 2.5* ^$$^ | 43 ± 3.6 |
| **SV (ul)** | 24.1 ± 2.3 | 29.4 ± 3 | 22.1 ± 0.9 | 20.7 ± 1^&^ |
| **CO (ml/min)** | 9.3 ± 1.2 | 12.8 ± 1.5 | 9.6 ± 0.3 | 8.9 ± 0.6^&^ |
| **LVw (mg)** | 114.7 ± 7.3 | 132.3 ± 7.8 | 84.1 ± 5.7^###^ ^$^ | 116.3 ± 4.6 |
| **cHW/BW (mg/g)** | 4.6 ± 0.3 | 5.1 ± 0.3 | 3.2 ± 0.2** ^####^ ^$$$^ | 5 ± 0.2 |
| **IVRT (ms)** | 25.6 ± 2.7 | 22.9 ± 1.2 | 18.6 ± 1.4 | 25.2 ± 4.5 |
| **IVCT (ms)** | 15.8 ± 1.8 | 13.4 ± 1.2 | 15.9 ± 2.6 | 17.4 ± 1.9 |
| **MVDT (ms)** | 24.7 ± 2.5 | 20.1 ± 2.4 | 21.2 ± 1.1 | 23.6 ± 1.7 |
| **AET (ms)** | 46.6 ± 2.4 | 42.9 ± 2.2 | 41.6 ± 1.1 | 40.9 ± 3.3 |
| **E/A** | 1.76 ± 0.17 | 1.42 ± 0.08 | 2.36 ± 0.2* ^###^ ^$$$^ | 1.42 ± 0.13 |

BW, body weight; HR, heart rate; IVSd, innerventricular septum in diastole; IVSs, innerventricular septum in systole; LVPWd, left ventricular posterior wall in diastole; LVPWs, left ventricular posterior wall in systole; LVIDd, left ventricular inner diameter in diastole; LVIDs, left ventricular inner diameter in systole; LVEF, left ventricular ejection fraction; LVFS, left ventricular fractional shortening; SV, stroke volume; CO, cardiac output; LVw, left ventricular weight; cHW/BW, calculated heart to body weight ratio; IVRT, isovolumic relaxation time; IVCT, isovolumic contraction time; MVDT, mitral valve deceleration time; AET, aortic ejection time; E/A, mitral valve E-wave to A-wave ratio. N=9 for WT and N2B splice recue, n=10 for RBM20-HET and N2B-KO for BW, HR, IVSs, IVSd, LVPWd, LVPWs, LVIDd, LVIDs, LV FS, LV FS, SV, CO, LVw and HW/BW. N=9 for WT and RBM20-HET, n=10 for N2B-KO and n=8 for N2B splice rescue for E/A, MV DT. N=7 for WT, n=9 for RBM20-HET, n=10 for N2B-KO and n=5 for N2B splice rescue for IVRT, IVCT, AET. Two-way ANOVA *, p≤0.05; **, p≤0.01; ***, p≤0.001; ****, p≤0.0001

* WT vs. N2B-KO; ^#^ RBM20-HET vs N2B-KO; ^&^ RBM20-HET vs. N2B splice rescue; ^$^ N2B splice rescue vs N2B-KO

## Supplemental Table 4: Catheter data

|  | **WT** | **RBM20-HET** | **N2B-KO** | **N2B splice rescue** |
| --- | --- | --- | --- | --- |
| **BW (g)** | 30.0 ± 1.3 | 29.6 ± 0.9 | 28.0 ± 0.7 | 26.9 ± 0.7 |
| **HR (BPM)** | 487 ± 14 | 464 ± 11 | 465 ± 15 | 473 ± 7 |
| **ESP art (mmHg)** | 103.5 ± 2.8 | 106.7 ± 3.8 | 113.2 ± 2.5 | 108.0 ± 4.1 |
| **EDP art (mmHg)** | 78.4 ± 4 | 80.6 ± 2.1 | 88.5 ± 1.1 | 83.3 ± 3.5 |
| **MAP (mmHg)** | 86.7 ± 3.4 | 89.3 ± 2.7 | 97.4 ± 1.4* | 91.5 ± 3.6 |
| **ESP (mmHg)** | 102.1 ± 3.5 | 100.9 ± 1.4 | 98.1 ± 3.7 | 99.2 ± 3.6 |
| **EDP (mmHg)** | 5.9 ± 0.7 | 6.3 ± 0.8 | 9.1 ± 1.3* ^#^ ^$^ | 5.0 ± 0.5 |
| **Pmax (mmHg)** | 107.9 ± 3.5 | 105.5 ± 1.3 | 104.5 ± 2.4 | 104.6 ± 3.1 |
| **Pmin (mmHg)** | 2.5 ± 0.7 | 2.5 ± 0.6 | 2.2 ± 0.5 | 2.2 ± 0.3 |
| **Pmean (mmHg)** | 45.1 ± 2.1 | 45.0 ± 1.8 | 41.5 ± 1.4 | 41.1 ± 2.3 |
| **Pdev (mmHg)** | 105.4 ± 3.2 | 103.1 ± 1.3 | 102.3 ± 2.3 | 102.4 ± 2.9 |
| **ESV (µL)** | 22.9 ± 3.2 | 32.7 ± 6.3 | 16.6 ± 1.2 | 26.5 ± 3.6 |
| **EDV (µL)** | 43.3 ± 5.6 | 55.6 ± 7.3 | 35.5 ± 2.7 | 42.1 ± 4.7 |
| **SV (µL)** | 24.7 ± 2.6 | 26.2 ± 1.2 | 22.4 ± 1.6 | 22.2 ± 2 |
| **CO (ml/min)** | 12.1 ± 1.5 | 11.5 ± 0.4 | 10.5 ± 1.0 | 10.4 ± 0.9 |
| **CI (ml/min/BW)** | 403 ± 42 | 397 ± 17 | 374 ± 32 | 398 ± 38 |
| **Ea (mmHg/µL)** | 4.5 ± 0.5 | 3.9 ± 0.2 | 4.7 ± 0.4 | 4.8 ± 0.6 |
| **TPR** | 7.9 ± 1.0 | 7.7 ± 0.3 | 9.3 ± 0.6 | 9.4 ± 1.1 |
| **EF (%)** | 57.4 ± 2.1 | 51.4 ± 4.5 | 65.1 ± 2.0^$^ | 52.9 ± 3.2 |
| **dP/dt max (mmHg/s)** | 7727 ± 438 | 7392 ± 432 | 8323 ± 470 | 7320 ± 408 |
| **-dP/dt max (mmHg/s)** | 9200 ± 478 | 7450 ± 584 | 9107 ± 584 | 8483 ± 514 |
| **SW (mmHg*mL)** | 2.2 ± 0.3 | 2.3 ± 0.1 | 1.9 ± 0.2 | 1.8 ± 0.1 |
| **Tauω (ms)** | 6.7 ± 0.3 | 7.4 ± 0.4 | 6.8 ± 0.5 | 6.8 ± 0.2 |
| **ESPVR** | 3.70 ± 0.82 | 3.76 ± 1.03 | 6.53 ± 1.27 | 4.73 ± 1.09 |
| **r^2^ (ESPVR)** | 0.972 ± 0.01 | 0.968 ± 0.01 | 0.963 ± 0.008 | 0.976 ± 0.006 |
| **EDPVR** | 0.132 ± 0.019 | 0.144 ± 0.023 | 0.304 ± 0.053** ^#^ ^$^ | 0.151 ± 0.031 |
| **r^2^ (EDPVR)** | 0.919 ± 0.05 | 0.932 ± 0.024 | 0.941 ± 0.032 | 0.946 ± 0.016 |
| **PRSW** | 58.4 ± 12.4 | 52.0 ± 7.2 | 73.3 ± 5.2 | 55.7 ± 6.8 |
| **r^2^ (PRSW)** | 0.941 ± 0.053 | 0.982 ± 0.009 | 0.982 ± 0.006 | 0.979 ± 0.008 |

BW, body weight; HR, heart rate; ESP art, arterial end-systolic pressure; EDP art, arterial end-diastolic pressure; MAP, mean arterial pressure; ESP, end-systolic pressure; EDP, end-diastolic pressure; Pmax, maximal pressure; Pmin, minimal pressure; Pmean, mean pressure; Pdev, developed pressure; ESV, end-systolic volume; EDV, end-diastolic volume; SV, stroke volume; CO, cardiac output; CI, cardiac index; Ea, arterial elastance; TPR, total peripheral resistance; EF, ejection fraction; SW, stroke work; Tauω, left ventricular relaxation time constant; ESPVR, end-systolic pressure volume relationship; r^2^ (ESPVR), ESPVR correlation coefficient; EDPVR, end-diastolic pressure volume relationship; r^2^ (EDPVR), EDPVR correlation coefficient; PRSW, preload recruitable stroke work; r^2^ (PRSW), PRSW correlation coefficient. N=8 for WT, n=10 for RBM20-HET and n=9 for N2B-KO and N2B splice rescue for BW, HR, ESP art, EDP art, MAP, ESP, EDP, Pmax, Pmin, Pmean, Pdev, ESV, EDV, SV, CO, CI, Ea, TPR, EF, dP/dt max, SW, -dP/dt max and Tauω. N=6 for WT, n=9 for RBM20-HET, n=8 for N2B-KO and N2B splice rescue for ESPVR. N=6 for WT, n=9 for RBM20-HETand N2B-KO, n=8 for N2B splice rescue for PRSW. N=7 for WT, RBM20-HET and N2B-KO, n=6 for for N2B splice rescue for EDPVR. Two-way ANOVA *, p≤0.05; **, p≤0.01

* WT vs. N2B-KO; ^#^ RBM20-HET vs N2B-KO; ^&^ RBM20-HET vs. N2B splice rescue; ^$^ N2B splice rescue vs N2B-KO

## Supplemental Table 5: Rbm20-dependent isoform expression

| **Gene** | **Relevance** | **Alternative Exons; Domains**^C^ |
| --- | --- | --- |
| Ank3 | Localizes to intercalated discs and Z-disc  [12] | E40-43; 2 lcr |
| Arhgap10 | Actin fiber formation; Z-disc after pressure overload [13] | E13-14; 1 Rho-Gap |
| Camk2d^A^,^B^ | Hemodynamic stress signaling [14] | E14-15; unstructured, 1 nls |
| Cflar | Regulates response to pressure overload [15] | E2; non coding |
| Fhl1 | Titin based mechanotransduction [16] | E1; start codon |
| Hmgb1 | Pressure overload induced cardiac hypertrophy [17, 18] | E1; non coding |
| Ldb3^A^,^B^ | Maintains Z-disc, cardiac hypertrophy [19] | E4-6/9; 1 ZASP-like motif, 1 lcr |
| Myh7^A^ | Myosin filament, hypertrophy [20] | E32-36; myosin-tail |
| Obscn^A^ | Sarcomere Z-disc and M-band [21] | E8-11/13-18/20-21/46; 13 IG |
| Ttc17 | Actin organization [22] | E17; unstructured |
| Ttn^A^,^B^ | Mechanotransduction, sarcomere organization [23] | I-band exons; several IG & PEVK |

^A^ mRNA bound by Rbm20 [24]. ^B^ Alternatively spliced in other RBM20 deficient species[11]. ^C^ Domain annotation at <http://smart.embl.de>

lcr = low complexity region; nls = nuclear localization signal

## Supplemental Table 6: Gene list for Figure 4C

| **GOterm** | **Associated genes** |
| --- | --- |
| regulation of fatty acid oxidation | Dgat2, Pparg, Ppargc1a |
| cellular response to cAMP | Hcn1, Hcn4, Nox4 |
| response to cold | Fos, Pparg, Ppargc1a |
| cellular response to fatty acid | Dgat2, Pparg, Ppargc1a |
| negative regulation of cellular carbohydrate metabolic process | Ppargc1a, Sik1, Sirt6 |

## Supplemental Table 7: Gene list for Figure 4D – reverted

| **GOterm – biological process** | **Associated genes** |
| --- | --- |
| regulation of fatty acid oxidation | Fos, Pparg, Ppargc1a, Dgat2 |
| negative regulation of carbohydrate metabolic process | Ppargc1a, Sik1, Sirt6 |
| skeletal muscle cell differentiation | Fos, Gpc1, Klhl40, Nr4a1 |
| cellular response to cAMP | Hcn1, Hcn4, Nox4 |
| positive regulation of muscle tissue development | Erbb3, Gpc1, Ppargc1a |
| **GOterm – cellular component** | **Associated genes** |
| respiratory chain | Cox6a2, Ndufb7, Ndufs7, Uqcr11, Uqcrq |
| oxidoreductase complex | Bckdha, Ndufb7, Ndufs7, Nox4, Uqcrq |
| **GOterm – molecular function** | **Associated genes** |
| growth factor binding | Erbb3, Gpc1, Pzp, Thbs1 |
| amino acid binding | Aars2, Ddah2, Thbs1 |
| oxidoreductase activity, acting on NAD(P)H | Ndufb7, Ndufs7, Nox4 |
| steroid hormone receptor binding | Nr4a1, Pparg, Ppargc1a |
| modified amino acid binding | Mgst1, Nox4, Thbs1 |

## Supplemental Table 8: Gene list for Figure 4D – misregulated

| **GOterm – biological process** | **Associated genes** |
| --- | --- |
| cell chemotaxis | Ccl12, Ccl6, Ccl8, Cd74, Cmklr1, Cxcl14, Edn3, Fcgr3, Flt1, Itga9, Itgb2, Myo9b, Nckap1l, Ptk2b, Serpine1, Spp1, Syk, Trpv4, Ephb1, Fgf16, Hbegf, Hmgb2, Hspb1, Kdr |
| cardiac cell development | Agt, Alpk3, Col14a1, Fhl2, Murc, Myh11, Nppa, Nppb, Pdlim5, Pi16, Prox1, Cacna1s, Flnc, Gpx1, Lmod2, Mypn |
| regulation of cell-substrate adhesion | Col8a1, Egflam, Fbln1, Fbln2, Kdr, Nid1, Ninj1, Ptk2b, S100a10, Spp1, Vwc2, Acer2, Bcl6, Dlc1, Postn, Serpine1, Agt, Bcl2l11, Itga11, Itgb2, Itgb6, Sorbs1 |
| positive regulation of epithelial cell migration | Agt, Alox12, Amot, Angpt1, Fgf16, Hspb1, Kdr, Prox1, Ptk2b, Aqp1, Hbegf, Efna1, Serpinf1 |
| other | Acta2, Adra1a, Agt, Alox12, Amot, Angpt1, Edn3, Gpx1, Nppa, Nppb, Nts, Per2, Prkg1, Ptgs1, Snta1, Trpv4, Acer2, Adh1, Aldh1a2, Aqp1, Eln, Ptk2b, Rbp4, Ret, Serpinf1, Tfrc, Twf2, Ace, Mcpt4, Postn, Rnls, Ankrd1, Bag3, Cradd, Fas, Kcnj2, Slc38a2, Ccl12, Ccl6, Ccl8, Gbp3, Gbp6, H2-Aa, H2-Ab1, H2-Eb1, Mrc1, Btg1, Cma1, Flt1, Hspb1, Itgb2, Kdr, Ptgis, Serpine1, Abcb4, Cd74, Ctss, Fcgr3, Hfe, Unc93b1, Csf1r, Fgfr3, Inpp5d, Itga9, Pth1r, Spp1, Syk |
| **GOterm – cellular component** | **Associated genes** |
| contractile fiber | Abra, Acta2, Adra1a, Ankrd1, Bag3, Cacna1s, Cryab, Fhl2, Flnc, Hspb1, Ky, Lmod2, Murc, Myh11, Myl4, Myl7, Mypn, Pdlim5, Scn5a, Tpm2, Xirp2, Nrap, Twf2 |
| extracellular matrix | Adamtsl2, Alpl, Angptl4, Bgn, Cilp, Clu, Cma1, Col14a1, Col1a2, Col4a4, Col4a5, Col8a1, Egflam, Eln, Fbln1, Fbln2, Fbn1, Gpc3, Lad1, Lama2, Mfap4, Mfap5, Mgp, Mmp11, Nid1, Postn, Serpine1, Serpinf1, Slc1a3, Spon2, Tgfbi, Tgm2, Tpsb2, Vwc2 |
| membrane raft | Abcb4, Angpt1, Cd14, Dlc1, Ephb1, Fas, Inpp5d, Insr, Itgb2, Kdr, Lrp8, Myof, Ptgis, Ptk2b, Ret, S100a10, Scn5a, Sele, Sorbs1 |
| sarcolemma | Adra1a, Alox12, Aqp1, Bgn, Cacna1s, Cacna2d1, Cacnb2, Clcn1, Dtna, Fas, Flnc, Kcnj2, Lama2, Scn5a, Slc38a2, Snta1, Trim72, Akap6, Ccdc109b, Micu1, Camk2b, Sln, Xdh, Scn4a, Scn4b |
| receptor complex | Acvr2b, Cd14, Cd74, Egf, Flt1, Hfe, Insr, Itga11, Itga9, Itgb2, Itgb6, Lrp8, Myh9, Plxna2, Pth1r, Ptk2b, Ptprn2, Ret, Sorbs1, Syk, Tfrc, Vwc2 |
| other | C1qa, C1qb, C1qc, Col14a1, Col1a2, Col4a4, Col4a5, Col8a1, P4ha2, Acta2, Enah, Ephb1, Myo9b, Trpv4, Twf2, Aqp1, Clic5, Cubn, Myh11, Myh9, Pth1r, Slc11a2, Slc38a2, Ush1c, Dtna, Epn3, Gnao1, Gnb3, Ptk2b, Rgs6, S100a10, S100a6, Syk, Sytl2, Clu, Dnajb2, Gpx1, Hspb7, Mecom, Park2, Dlc1, Dstn, Hfe, Spta1, Ackr3, Ap4b1, Sele, Tfrc, Agt, Angptl4, Cfh, Tmprss13, Zbtb38, Amot, Cryab, Sorbs1, Gck, Sept6 |
| **GOterm – molecular function** | **Associated genes** |
| gated channel activity | Ano10, Aqp1, Cacna1s, Cacna2d1, Cacnb2, Clcn1, Clcn6, Clic5, Cngb1, Kcnj2, Kcnn1, P2rx5, Ptk2b, Scn4a, Scn4b, Scn5a, Slc17a7, Trpv4 |
| actin binding | Abra, Ace, Adssl1, Cnn1, Dstn, Enah, Flnc, Lmod2, Msrb1, Mtss1, Myh11, Myh9, Myl4, Myo9b, Mypn, Nrap, Park2, Pdlim5, Ppp1r42, Snta1, Spta1, Tpm2, Trpv4, Twf2, Ush1c, Xirp2 |
| organic acid binding | Aars, Alox5ap, Fabp7, Glul, Insr, P4ha2, Pcx, Pygl, Sele, Slc1a3, Sytl2, Trim72 |
| transmembrane receptor protein kinase activity | Acvr2b, Col1a2, Fgfr3, Flt1, Il10ra, Insr, Kdr, Scn5a, Csf1r, Ephb1, Ret |
| symporter activity | Slc11a2, Slc16a1, Slc16a5, Slc16a7, Slc17a7, Slc1a3, Slc1a4, Slc38a1, Slc38a2, Slc38a3 |
| anion channel activity | Ano10, Clcn1, Clcn6, Clic5, Slc17a7, Slc1a4 |
| other | Arntl, Dnajb2, Npas2, Park2, Pttg1, Tfrc, Ahsa1, Bag3, Dnajb4, Dnajb5, Camk2b, Cnn1, Kcnn1, Mknk2, Myh11, Myh9, Myo9b, Scn5a, Snta1, Trpv4, Abcg1, Ap4b1, Kpna2, Tomm5, Unc93b1, Fbln1, Fbn1, Itgb6, Kdr, Sema7a, Syk, Abca12, Abca4, Abcb4, Atp8a2, Plscr5, Rbp4, Aqp1, Aqp8, Slc16a1, Slc16a5, Slc16a7, Dct, Gm1840, Hsd3b3, Ptgis, Qsox1, Tmx1, Adam19, Cttnbp2, Enah, Gpx1, Inpp5d, Mypn, Sh3bgr, Aldh1a2, Cubn, Ogdhl, Opn4, P4ha2, Pcx, Pygl, Ackr3, Acvr2b, Cd74, Crlf1, Csf1r, Ifngr1, Il10ra, Gfra2, Noct |

## Supplemental Table 9: Gene list for Figure S3 – RBM20-HET

| **GOterm – biological process** | **Associated genes** |
| --- | --- |
| regulation of relaxation of cardiac muscle | Camk2d, Ttn |
| positive regulation of interferon-alpha production | Hmgb1, Tlr4 |
| gas transport | Hba-a1, Hba-a2 |
| mismatch repair | Hmgb1, Msh3 |
| adult heart development | Myh7, Ttn |
| astrocyte development | Lamb2, Tlr4 |
| skeletal myofibril assembly | Cflar, Ttn |
| **GOterm – cellular component** | **Associated genes** |
| haptoglobin-hemoglobin complex | Hba-a1, Hba-a2, Rps14, Rps27a |
| T-tubule | Cacna2d1, Camk2d, Dysf, Micu1 |
| muscle myosin complex | Myh7, Ttn |
| axon initial segment | Ank3, Camk2d |
| **GOterm – molecular function** | **Associated genes** |
| haptoglobin binding | Hba-a1, Hba-a2 |
| alpha-actinin binding | Ldb3, Synpo2, Ttn |
| muscle alpha-actinin binding | Ldb3, Synpo2, Ttn |
| DNA secondary structure binding | Hmgb1, Msh3 |
| lipopolysaccharide binding | Hmgb1, Tlr4 |
| enoyl-CoA hydratase activity | Auh, Hadha |
| peptidase activator activity | Cflar, Vcp |

## Supplemental Table 10: Gene list for Figure S3 – Splice rescue

| **GOterm – biological process** | **Associated genes** |
| --- | --- |
| myofibril assembly | Camk2d, Myh6, Myh7, Pdlim5, Ttn, Myl2, Camk2d, Tpm1, Cflar, Ldb3 |
| positive regulation of nucleoside metabolic process | Entpd5, Park7, Vcp |
| erythrocyte development | Ank1, Hba-a1, Hba-a2 |
| **GOterm – cellular component** | **Associated genes** |
| muscle myosin complex | Myh6, Myh7, Myl2, Obscn, Ttn, Tpm1 |
| respiratory chain complex | Cox7b, Ndufv1, Ndufv3, Park7 |
| cytosolic small ribosomal subunit | Hba-a1, Hba-a2, Rps14, Rps27a, Rps3a1 |
| axon initial segment | Ank3, Camk2d |
| **GOterm – molecular function** | **Associated genes** |
| actinin binding | Obscn, Ttn, Camk2d, Ldb3, Pdlim5 |
| haptoglobin binding | Hba-a1, Hba-a2, Park7 |
| enoyl-CoA hydratase activity | Auh, Hadha, Park7, Ivd |
| ubiquitin-specific protease binding | Cflar, Park7, Vcp |
| microfilament motor activity | Myh6, Myh7 |
| mismatched DNA binding | Msh3, Tdg |

# Supplemental References

1. Radke MH, Peng J, Wu Y, McNabb M, Nelson OL, Granzier H, Gotthardt M (2007) Targeted deletion of titin N2B region leads to diastolic dysfunction and cardiac atrophy. ProcNatlAcadSciUSA 104:3444–3449.

2. Methawasin M, Hutchinson KR, Lee E-J, Smith JE, Saripalli C, Hidalgo CG, Ottenheijm CAC, Granzier H (2014) Experimentally increasing titin compliance in a novel mouse model attenuates the Frank-Starling mechanism but has a beneficial effect on diastole. Circulation 129:1924–1936. doi: 10.1161/CIRCULATIONAHA.113.005610

3. Warren CM, Krzesinski PR, Greaser ML (2003) Vertical agarose gel electrophoresis and electroblotting of high-molecular-weight proteins. Electrophoresis 24:1695–1702.

4. Livak KJ, Schmittgen TD (2001) Analysis of relative gene expression data using real-time quantitative PCR and the 2(-Delta Delta C(T)) Method. Methods San Diego Calif 25:402–408. doi: 10.1006/meth.2001.1262

5. Granzier HL, Radke MH, Peng J, Westermann D, Nelson OL, Rost K, King NMP, Yu Q, Tschöpe C, McNabb M, et al (2009) Truncation of titin’s elastic PEVK region leads to cardiomyopathy with diastolic dysfunction. Circ Res 105:557–564. doi: 10.1161/CIRCRESAHA.109.200964

6. Dodt M, Roehr JT, Ahmed R, Dieterich C (2012) FLEXBAR-Flexible Barcode and Adapter Processing for Next-Generation Sequencing Platforms. Biology 1:895–905. doi: 10.3390/biology1030895

7. Dobin A, Davis CA, Schlesinger F, Drenkow J, Zaleski C, Jha S, Batut P, Chaisson M, Gingeras TR (2013) STAR: ultrafast universal RNA-seq aligner. Bioinforma Oxf Engl 29:15–21. doi: 10.1093/bioinformatics/bts635

8. Trapnell C, Roberts A, Goff L, Pertea G, Kim D, Kelley DR, Pimentel H, Salzberg SL, Rinn JL, Pachter L (2012) Differential gene and transcript expression analysis of RNA-seq experiments with TopHat and Cufflinks. Nat Protoc 7:562–578. doi: 10.1038/nprot.2012.016

9. Schafer S, Miao K, Benson CC, Heinig M, Cook SA, Hubner N (2015) Alternative Splicing Signatures in RNA-seq Data: Percent Spliced in (PSI). Curr Protoc Hum Genet Editor Board Jonathan Haines Al 87:11.16.1-11.16.14. doi: 10.1002/0471142905.hg1116s87

10. Pertea M, Pertea GM, Antonescu CM, Chang T-C, Mendell JT, Salzberg SL (2015) StringTie enables improved reconstruction of a transcriptome from RNA-seq reads. Nat Biotechnol 33:290–295. doi: 10.1038/nbt.3122

11. Guo W, Schafer S, Greaser ML, Radke MH, Liss M, Govindarajan T, Maatz H, Schulz H, Li S, Parrish AM, et al (2012) RBM20, a gene for hereditary cardiomyopathy, regulates titin splicing. Nat Med 18:766–773. doi: 10.1038/nm.2693

12. Yamankurt G, Wu HC, McCarthy M, Cunha SR (2015) Exon organization and novel alternative splicing of Ank3 in mouse heart. PloS One 10:e0128177. doi: 10.1371/journal.pone.0128177

13. Borges L, Bigarella CL, Baratti MO, Crosara-Alberto DP, Joazeiro PP, Franchini KG, Costa FF, Saad STO (2008) ARHGAP21 associates with FAK and PKCzeta and is redistributed after cardiac pressure overload. Biochem Biophys Res Commun 374:641–646. doi: 10.1016/j.bbrc.2008.07.085

14. Backs J, Backs T, Neef S, Kreusser MM, Lehmann LH, Patrick DM, Grueter CE, Qi X, Richardson JA, Hill JA, et al (2009) The delta isoform of CaM kinase II is required for pathological cardiac hypertrophy and remodeling after pressure overload. Proc Natl Acad Sci U S A 106:2342–2347. doi: 10.1073/pnas.0813013106

15. Giampietri C, Petrungaro S, Musumeci M, Coluccia P, Antonangeli F, De Cesaris P, Filippini A, Marano G, Ziparo E (2008) c-Flip overexpression reduces cardiac hypertrophy in response to pressure overload. J Hypertens 26:1008–1016. doi: 10.1097/HJH.0b013e3282f6a179

16. Sheikh F, Raskin A, Chu P-H, Lange S, Domenighetti AA, Zheng M, Liang X, Zhang T, Yajima T, Gu Y, et al (2008) An FHL1-containing complex within the cardiomyocyte sarcomere mediates hypertrophic biomechanical stress responses in mice. J Clin Invest 118:3870–80. doi: 10.1172/JCI34472

17. Zhu X, Messer JS, Wang Y, Lin F, Cham CM, Chang J, Billiar TR, Lotze MT, Boone DL, Chang EB (2015) Cytosolic HMGB1 controls the cellular autophagy/apoptosis checkpoint during inflammation. J Clin Invest 125:1098–1110. doi: 10.1172/JCI76344

18. Zhang L, Liu M, Jiang H, Yu Y, Yu P, Tong R, Wu J, Zhang S, Yao K, Zou Y, et al (2016) Extracellular high-mobility group box 1 mediates pressure overload-induced cardiac hypertrophy and heart failure. J Cell Mol Med 20:459–470. doi: 10.1111/jcmm.12743

19. Lin X, Ruiz J, Bajraktari I, Ohman R, Banerjee S, Gribble K, Kaufman JD, Wingfield PT, Griggs RC, Fischbeck KH, et al (2014) Z-disc-associated, alternatively spliced, PDZ motif-containing protein (ZASP) mutations in the actin-binding domain cause disruption of skeletal muscle actin filaments in myofibrillar myopathy. J Biol Chem 289:13615–13626. doi: 10.1074/jbc.M114.550418

20. Morita H, Rehm HL, Menesses A, McDonough B, Roberts AE, Kucherlapati R, Towbin JA, Seidman JG, Seidman CE (2008) Shared genetic causes of cardiac hypertrophy in children and adults. N Engl J Med 358:1899–1908. doi: 10.1056/NEJMoa075463

21. Sanger JW, Sanger JM (2001) Fishing out proteins that bind to titin. JCell Biol 154:21–24.

22. Bontems F, Fish RJ, Borlat I, Lembo F, Chocu S, Chalmel F, Borg J-P, Pineau C, Neerman-Arbez M, Bairoch A, et al (2014) C2orf62 and TTC17 are involved in actin organization and ciliogenesis in zebrafish and human. PloS One 9:e86476. doi: 10.1371/journal.pone.0086476

23. Gautel M, Djinović-Carugo K (2016) The sarcomeric cytoskeleton: from molecules to motion. J Exp Biol 219:135–145. doi: 10.1242/jeb.124941

24. Maatz H, Jens M, Liss M, Schafer S, Heinig M, Kirchner M, Adami E, Rintisch C, Dauksaite V, Radke MH, et al (2014) RNA-binding protein RBM20 represses splicing to orchestrate cardiac pre-mRNA processing. J Clin Invest 124:3419–3430. doi: 10.1172/JCI74523
